# Genomic and immune heterogeneity are associated with differential responses to therapy in melanoma

**DOI:** 10.1038/s41525-017-0013-8

**Published:** 2017-04-07

**Authors:** Alexandre Reuben, Christine N. Spencer, Peter A. Prieto, Vancheswaran Gopalakrishnan, Sangeetha M. Reddy, John P. Miller, Xizeng Mao, Mariana Petaccia De Macedo, Jiong Chen, Xingzhi Song, Hong Jiang, Pei-Ling Chen, Hannah C. Beird, Haven R. Garber, Whijae Roh, Khalida Wani, Eveline Chen, Cara Haymaker, Marie-Andrée Forget, Latasha D. Little, Curtis Gumbs, Rebecca L. Thornton, Courtney W. Hudgens, Wei-Shen Chen, Jacob Austin-Breneman, Robert Szczepaniak Sloane, Luigi Nezi, Alexandria P. Cogdill, Chantale Bernatchez, Jason Roszik, Patrick Hwu, Scott E. Woodman, Lynda Chin, Hussein Tawbi, Michael A. Davies, Jeffrey E. Gershenwald, Rodabe N. Amaria, Isabella C. Glitza, Adi Diab, Sapna P. Patel, Jianhua Hu, Jeffrey E. Lee, Elizabeth A. Grimm, Michael T. Tetzlaff, Alexander J. Lazar, Ignacio I. Wistuba, Karen Clise-Dwyer, Brett W. Carter, Jianhua Zhang, P. Andrew Futreal, Padmanee Sharma, James P. Allison, Zachary A. Cooper, Jennifer A. Wargo

**Affiliations:** 1 Department of Surgical Oncology, The University of Texas MD Anderson Cancer Center, 1515 Holcombe Blvd, Houston, TX 77030 USA; 2 Department of Genomic Medicine, The University of Texas MD Anderson Cancer Center, 1515 Holcombe Blvd, Houston, TX 77030 USA; 3 Department of Cancer Biology, The University of Texas MD Anderson Cancer Center, 1515 Holcombe Blvd, Houston, TX 77030 USA; 4 Department of Translational Molecular Pathology, The University of Texas MD Anderson Cancer Center, 1515 Holcombe Blvd, Houston, TX 77030 USA; 5 Department of Pathology, The University of Texas MD Anderson Cancer Center, 1515 Holcombe Blvd, Houston, TX 77030 USA; 6 Department of Stem Cell Transplantation, The University of Texas MD Anderson Cancer Center, 1515 Holcombe Blvd, Houston, TX 77030 USA; 7 Department of Melanoma Medical Oncology, The University of Texas MD Anderson Cancer Center, 1515 Holcombe Blvd, Houston, TX 77030 USA; 8 Department of Cancer Medicine, The University of Texas MD Anderson Cancer Center, 1515 Holcombe Blvd, Houston, TX 77030 USA; 9 Department of Biostatistics, The University of Texas MD Anderson Cancer Center, 1515 Holcombe Blvd, Houston, TX 77030 USA; 10 Department of Diagnostic Radiology, The University of Texas MD Anderson Cancer Center, 1515 Holcombe Blvd, Houston, TX 77030 USA; 11 Department of Immunology, The University of Texas MD Anderson Cancer Center, 1515 Holcombe Blvd, Houston, TX 77030 USA; 12 Department of Genitourinary Medical Oncology, The University of Texas MD Anderson Cancer Center, 1515 Holcombe Blvd, Houston, TX 77030 USA; 13 Present Address: MedImmune, Gaithersburg, MD 20878 USA

## Abstract

Appreciation for genomic and immune heterogeneity in cancer has grown though the relationship of these factors to treatment response has not been thoroughly elucidated. To better understand this, we studied a large cohort of melanoma patients treated with targeted therapy or immune checkpoint blockade (*n* = 60). Heterogeneity in therapeutic responses via radiologic assessment was observed in the majority of patients. Synchronous melanoma metastases were analyzed via deep genomic and immune profiling, and revealed substantial genomic and immune heterogeneity in all patients studied, with considerable diversity in T cell frequency, and few shared T cell clones (<8% on average) across the cohort. Variables related to treatment response were identified via these approaches and through novel radiomic assessment. These data yield insight into differential therapeutic responses to targeted therapy and immune checkpoint blockade in melanoma, and have key translational implications in the age of precision medicine.

## Melanoma: Tumor differences within a patient may explain heterogeneous responses

Patients with metastatic melanoma display molecular and immune differences across tumor sites associated with differential drug responses. A team led by Jennifer Wargo from the University of Texas MD Anderson Cancer Center, Houston, USA, studied the radiological responses of 60 patients with metastatic melanoma, half of whom received targeted drug therapy and half of whom received an immune checkpoint inhibitor. The majority (83%) showed differences in responses across metastases. The group then profiled tumors in a subset, and found molecular and immune heterogeneity in different tumors within the same patient. Heterogeneity in mutational and immune profiles within tumors from individual patients could explain differences in treatment response. Knowing this, the authors emphasize the importance of acquiring biopsies from more than one tumor site in order to best tailor therapies to the features of metastatic cancer.

## Introduction

Major breakthroughs in melanoma treatment have been made through the use of targeted therapy^1^ and immune checkpoint blockade.^2, 3^ However, responses are heterogeneous and are not always durable.^4^ Interest in the role of tumor genomic and immune heterogeneity in primary, metastatic,^5^ and synchronous tumors has grown,^6^ yielding mechanistic insight into clonal evolution,^7^ the seeding of metastases,^7^ tumor escape,^6^ and differential responses to therapy.^5, 6, 8, 9^ However, the relationship between genomic and immune heterogeneity and link to tumor growth and response to therapy has not been extensively studied.

To date, primary assessment of tumor heterogeneity has been performed on tissue derived from primary tumors or metastases. Groundbreaking work in genomics has established phylogenetic trees based on differential whole exome sequencing (WES) analyses of multiple regions within a single tumor, yielding insight into clonal evolution of various solid tumors.^7, 9^ Additional studies have built on this concept and compared primary to metastatic tumors,^7, 9^ differentiated synchronous metastases,^5, 7^ and studied evolutionary changes in response to treatment.^5, 6^ More recent studies have also highlighted the presence and importance of immune heterogeneity and how it may relate to treatment response.^10^

In addition to study of solid tumor at the site of a metastasis, assessment of tumor heterogeneity has been performed using less invasive liquid biopsy approaches on ctDNA (circulating tumor DNA) and tumor-derived exosomes^11–13^ that carry the potential advantage of capturing mutational profiles from multiple tumor deposits.^11^ ctDNA assessment has also been used to identify presence of multiple concurrent tumor resistance mechanisms, study clonal evolution with treatment,^12^ and provide early detection of new mutations even prior to gross tumor growth.^13^

More recently, less invasive radiomic approaches have been developed based on novel analysis of conventional imaging studies, such as computed tomography (CT),^14, 15^ magnetic resonance imaging (MRI), and positron emission tomography (PET). Radiomics refers to the high-throughput extraction of numerous quantitative features from routine imaging examinations, and the utilization of such an approach is somewhat nascent in assessing response to melanoma-targeted therapy and immunotherapy.^16^ The concept of textural analysis was first introduced in 1973 by Haralick and colleagues.^17^ In radiomics, texture analyses can be used to provide a measure of intratumoral heterogeneity.^15^ This technique has been employed to capture and evaluate the imaging data of a wide variety of solid tumors, including gliomas and lung, breast, colon, hepatocellular, and renal cell cancers,^15^ among others, to predict responses to various cancer treatments, provide prognostic information,^18, 19^ identify tumor histology,^18^ and even predict cancer-associated mutations and gene expression signatures.^19^

In this study, we sought to assess the relevance and relationship of genomic and immune heterogeneity to therapeutic responses in multiple melanoma metastases from patients on targeted therapy or immune checkpoint blockade as well as treatment-naïve patients at clinical presentation. Importantly, we also incorporated novel radiomic assessment of heterogeneity in synchronous metastases, as well as its relationship to therapeutic response. Through these studies, we identified significant heterogeneity in clinical responses of synchronous metastases to targeted therapy and immune checkpoint blockade in the majority of patients studied. Substantial genomic and immune heterogeneity in synchronous metastases of patients on targeted therapy or immune checkpoint blockade was also observed, and this was also seen in synchronous metastases in a smaller cohort of treatment-naïve patients, suggesting that this was not simply a result of selective pressure from therapy. Though these findings need to be validated in other histologies, these studies have potentially far-reaching implications.

## Results

### Heterogeneity in therapeutic responses to targeted therapy and immune checkpoint blockade is consistently observed

**Fig. 1:**
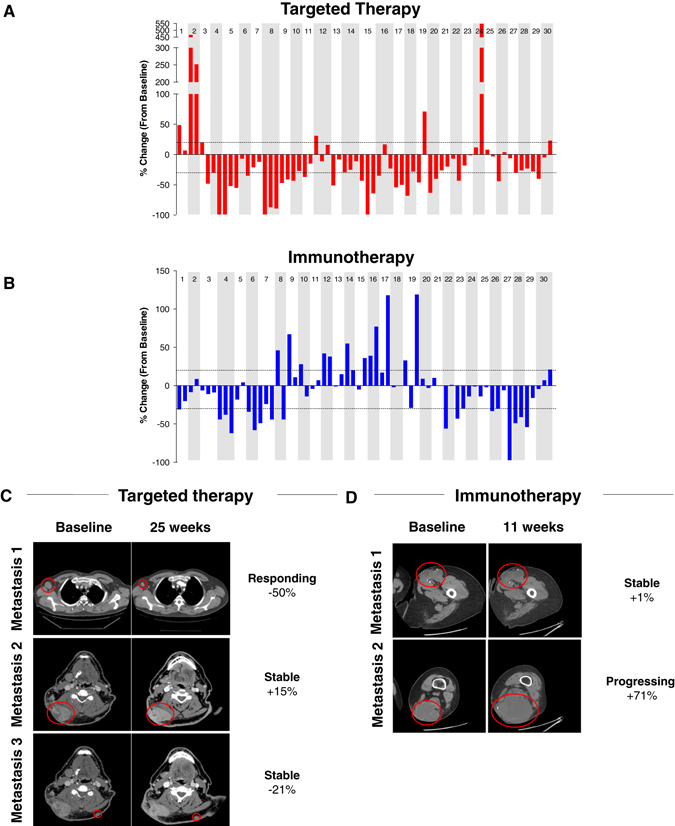
Differential intrapatient responses to targeted therapy and immune checkpoint blockade are widespread in patients with synchronous melanoma metastases. **a** Change in tumor size from baseline in a cohort of 30 patients with synchronous melanoma metastases treated with BRAF/MEK-inhibitor combination first-line therapy. **b** Change in tumor size from baseline in a cohort of 30 patients with synchronous melanoma metastases treated with PD-1 checkpoint blockade first-line therapy. Representative CT scans showing differential intrapatient responses to therapy in two patients treated with **c** BRAF-inhibitor therapy and **d** PD-1 blockade

To gain insight into the degree of clinical heterogeneity observed in therapeutic responses, we first analyzed radiographic responses of synchronous melanoma metastases to therapy in a large cohort of patients with metastatic melanoma (*n* = 60) that received first-line treatment with either targeted therapy with combined BRAF and MEK inhibitors or immune checkpoint blockade with anti-programmed cell death (PD)-1-based therapy (*n* = 30 per group). Dimensions of synchronous metastases were measured at baseline and at first re-staging as described,^20^ and change in tumor size was calculated based on percent change from baseline. In these studies, heterogeneity in therapeutic responses was observed in the vast majority of patients studied (83% of patients on targeted therapy and on immune checkpoint blockade) with differences of 10% or more when comparing multiple synchronous metastases within each given patient, with a median difference in tumor growth of 28 and 23% for patients treated with targeted therapy and immune checkpoint blockade, respectively (Fig. 1a, b, Supplementary Table S1A-B, and Supplementary Table S2). Importantly, intrapatient heterogeneity in clinical responses was also observed when considering more stringent thresholds using 20 and 50% intrapatient differences, as well as changes in response status and RECIST class (Supplementary Table S2). Of note, 23% of targeted therapy patients and 20% of immune checkpoint blockade patients presented differences in rate of tumor growth greater than 50%, suggesting prominent therapeutic heterogeneity affects a considerable proportion of patients. Representative case examples are shown for patients on targeted therapy and immune checkpoint blockade (Fig. 1c, d).

### Significant genomic heterogeneity exists in synchronous melanoma metastases during (and prior to) treatment with targeted therapy and immune checkpoint blockade

Considering the heterogeneity in therapeutic responses to both targeted therapy and immune checkpoint blockade, and knowledge gained from pre-existing literature,^5^ we next investigated the role of genomic heterogeneity in synchronous tumors from a subset of these patients who underwent resection of multiple metastases for therapeutic purposes (*n* = 33 tumors from 15 patients). This included patients who were treated with targeted therapy or immune checkpoint blockade, as well as several patients who were naïve to systemic treatment (Supplementary Fig. S1A-S15A and Supplementary Table S3).

**Fig. 2:**
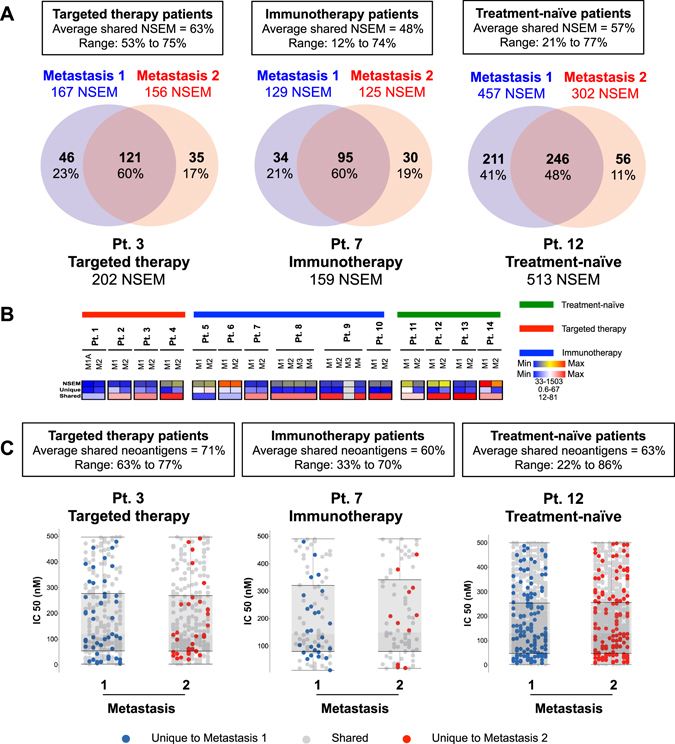
Molecular heterogeneity in synchronous melanoma metastases. **a** Overall mutational analysis and overlap in targeted therapy, immune checkpoint blockade, and treatment-naïve patients and representative patients from each treatment background presented as percentage of shared (*purple*) and unique (*blue* and *red*) NSEM between synchronous metastases. **b** Aggregate genomic data showing number of somatic NSEM, percent unique and shared in targeted therapy, immune checkpoint blockade, and treatment-naïve patients. **c** Predicted neoantigens in a representative targeted therapy, immune checkpoint blockade, and treatment-naïve patients based on their respective IC50 values. Shown are neoantigens shared (*gray*) and unique (*blue* or *red*) in each metastasis within patients presenting an IC50 < 500 nM

In these studies, genomic heterogeneity existed in all patients studied, with roughly half of non-synonymous exonic mutations (NSEM) shared between synchronous tumors across each of the cohorts (mean shared % of NSEMs of 63, 48, 57 in targeted therapy, checkpoint blockade, and treatment-naïve groups) with representative examples and aggregate data shown (Fig. 2a, b, Supplementary Fig. S1B-S15B, and Supplementary Table S4). Mutational signatures were also assessed suggesting that, on average, 79% were C-to-T ultraviolet (UV)-associated, as expected (Supplementary Fig. S1C-S15C).^21, 22^ Mutations in driver genes such as *BRAF* were also detected in 5/14 of patients (36%), as expected, and conserved across synchronous metastases, in-line with previous reports (Supplementary Fig. S16).^5, 23^ Intrapatient heterogeneity was not as profound as interpatient heterogeneity, in-line with published data (Supplementary Table S5).^5, 9, 21–34^

Given the increasing appreciation for the role of neoantigens in responses to therapy,^35^ we next performed in silico analysis of putative neoantigens in our cohort from WES data using the NetMHC3.4 algorithm based on patient-restricted HLA-A alleles (Supplementary Table S3).^36^ These studies demonstrated that though a considerable number of neoantigens are shared between synchronous metastases across the cohort of patients (mean shared neoantigens = 71% for targeted therapy, 60% for immune checkpoint blockade, and 63% for treatment-naïve patients), there are also a large number of neoantigens unique to synchronous metastases, including neoantigens predicted to bind HLA-A with high affinity (IC50 < 100 nM, Fig. 2c, Supplementary Fig. S1D-S15D, and Supplementary Table S4), which could differentially impact tumor immunogenicity.

### Significant immune heterogeneity also exists in synchronous melanoma metastases during (and prior to) treatment with targeted therapy and immune checkpoint blockade

Considering the genomic heterogeneity observed within these patients and its capacity to influence tumor immunogenicity and microenvironment, we next sought to investigate immune heterogeneity within this unique cohort via flow cytometry, immunohistochemistry (IHC), T cell receptor sequencing, and gene expression profiling.

**Fig. 3:**
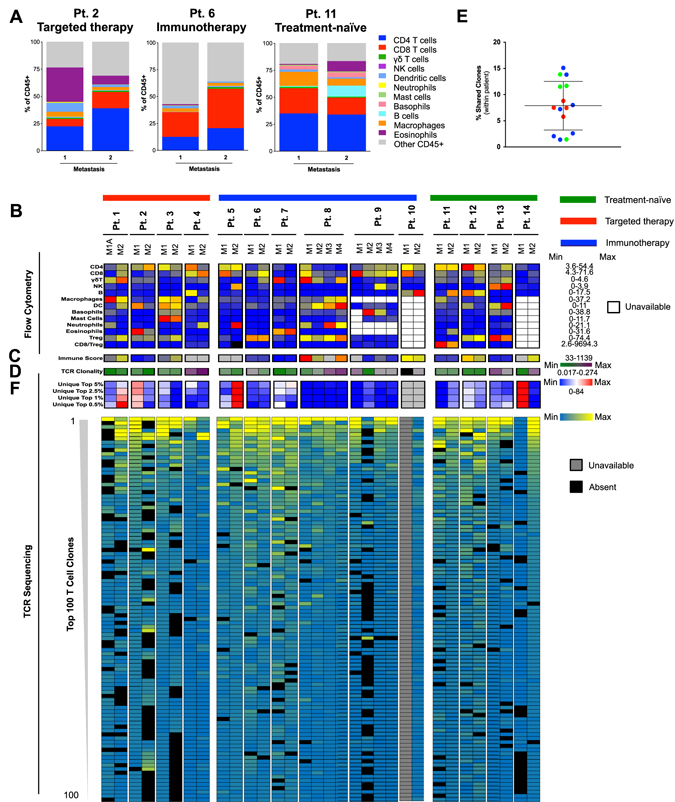
Immune heterogeneity in synchronous melanoma metastases. **a** Flow cytometry demonstrating the relative contribution of each immune cell subset as a percentage of total CD45^+^ cells within synchronous metastases in a representative targeted therapy, immune checkpoint blockade, and treatment-naïve patient. **b** Aggregate flow cytometric profiling data for all targeted therapy, immune checkpoint blockade, and treatment-naïve patients. **c** Immune score as calculated from gene expression profiling data. **d** Aggregate data showing TCR clonality in each metastasis. **e** Aggregate TCR sequencing data showing the percent of shared T cells detected in synchronous metastases within each patient as a % of total T cell clones. *Center value* represents mean and *error bar* represents SD. **f** Aggregate TCR sequencing data showing unique T cell clones within the top 5, 2.5, 1, 0.5% and 100 most prevalent T cell clones per patient

We first quantified infiltrating immune cell subsets such as T cells, γδ T cells, B cells, natural killer (NK) cells, dendritic cells, macrophages, mast cells, neutrophils, eosinophils, and basophils by flow cytometry, as previously described.^37^ These immune cell subsets were plotted based on their relative frequency as a percentage of CD45^+^ immune cells in each metastasis, and revealed marked differences in the relative abundance of immune cell subsets in synchronous metastases of all patients studied. Representative data for each treatment group are shown (Fig. 3a), as is a summary of aggregate data (Fig. 3b). Of note, the largest and most consistent differences were observed in CD4^+^ (79% of patients), CD8^+^ (43% of patients), and regulatory T cell (Treg, 50% of patients) frequency (Fig. 3b, Supplementary Fig. S1E–S15E, and Supplementary Table S6). Heterogeneity in CD4, CD8, and Treg infiltration was corroborated via IHC (Supplementary Fig. S1F-S15F).

We next investigated components of the tumor microenvironment via gene expression profiling. In these studies, we observed unique gene expression profiles in synchronous metastases in all patients studied, with differences in the immune score of synchronous metastases based on expression of cytokines and chemokines, human leukocyte antigen (HLA) molecules, adhesion molecules and interferon (IFN) pathway genes (Fig. 3c, and Supplementary Table S7).^38, 39^

To better understand the antigen specificity of tumor infiltrating T cells, we performed sequencing of the CDR3 region of the T cell receptor. Initial assessment of these data suggested large changes in T cell clonality, with substantial intrapatient heterogeneity in T cell clones (Fig. 3d, and Supplementary Fig. S1G–S15G), and less than 8% of total T cell clones shared between synchronous tumors, on average (range: 1.4–15.1% shared clones, Fig. 3e). Importantly, we studied the heterogeneity present in the top 5.0, 2.5, 1.0, 0.5%, as well as top 100 T-cell clones, and again observed striking heterogeneity, with many high-frequency clones being entirely restricted to individual metastases within the same patient (Fig. 3f). Interestingly, Spearman-rank correlations investigating the top 10 and top 100 clones revealed little correlation between metastases, suggesting a different T cell hierarchy may exist across synchronous metastases (Supplementary Fig. S17). Strikingly, in 7/14 patients (50%), at least 1 (and up to 5) of the top 10 most prevalent T cell clones were entirely restricted to individual metastases (Fig. 3f), suggesting that significant heterogeneity in antigen-specific T cell responses could affect synchronous melanoma metastases.

### Genomic and immune heterogeneity in synchronous metastases contribute to differential responses to therapy

**Fig. 4:**
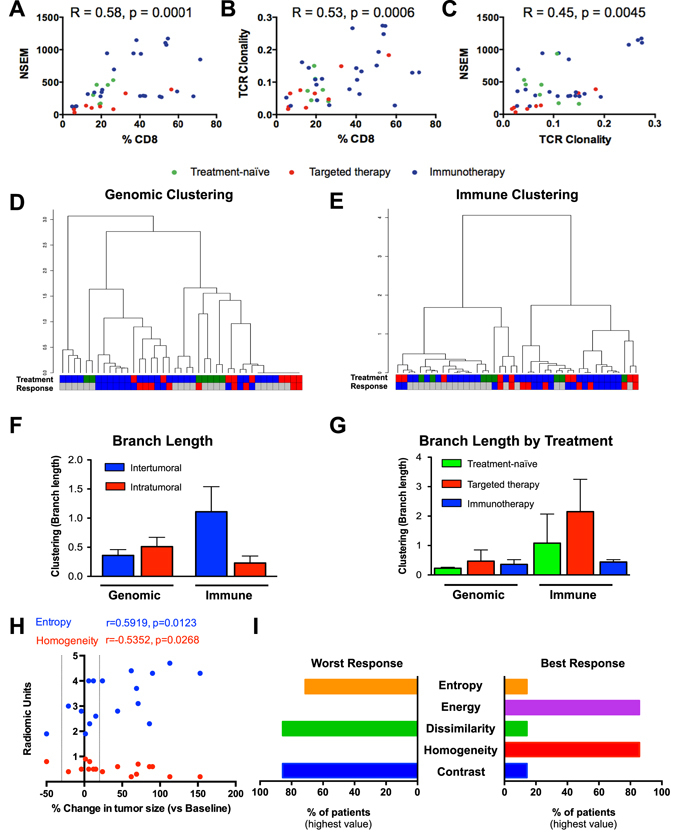
Genomic and immune heterogeneity are associated with differential tumor growth, and response to targeted therapy and immune checkpoint blockade. Genomic and immune data were studied, and **a** somatic NSEM and CD8%, **b** CD8% and TCR clonality and **c** somatic NSEM and TCR clonality were plotted to show correlation. Agglomerative Ward’s hierarchical clustering of individual samples based on genomic (**d**) and immune (**e**) parameters. **f** Average clustering branch length of genomic and immune parameters of different regions of the same metastasis (*red*) and different metastases in the same patient (*blue*). **g** Average clustering branch length of genomic and immune parameters based on treatment background. **h** Correlation between radiographic change in tumor size from baseline and radiomic texture analysis features such as entropy (*blue*) and homogeneity (*red*). **i** Percentage of patients in whom the best and worst responding synchronous lesions within each patient show the highest entropy, energy, dissimilarity, homogeneity, and contrast by texture analysis

Recent studies have suggested that certain molecular and immune features of a tumor may shape responses to therapy in melanoma and other cancers.^35, 40^ To better understand the relationship of the observed heterogeneous clinical responses with molecular and immune features within synchronous metastatic tumors, we performed an integrated analysis of genomic and immune features studied in our cohort as they relate to therapeutic response. We first performed pairwise comparisons between selected molecular and immune markers, and found a statistically-significant correlation between mutational load (NSEM) and CD8^+^ T cell frequency (Fig. 4a, *r* = 0.58, *p* = 0.0001 and Supplementary Fig. S1H–S15H), suggesting that a higher mutational burden is associated with enhanced CD8^+^ T cell infiltrate. We next compared T-cell receptor (TCR) clonality and CD8^+^ T cell frequency, and observed a strong correlation as well (Fig. 4b, *r* = 0.53, *p* = 0.0006 and Supplementary Fig. S1H-S15H), implying that more clonal T cell responses are associated with an enhanced CD8^+^ T cell infiltrate. We then compared TCR clonality to the mutational burden, and demonstrated that a higher mutational burden was associated with a more clonal T cell infiltrate (i.e., a less diverse T cell repertoire) (Fig. 4c, *r* = 0.45, *p* = 0.0045 and Supplementary Fig. S1H–S15H). These data are provocative, and suggest that rather than inducing antigen-specific responses against a broad range of neoantigens, a high mutational burden may actually enhance the likelihood of generating immunogenic epitopes capable of inducing an antigen-specific response.^41^

We next went back to genomic and immune profiling data to elucidate molecular and immunologic parameters associated with differential responses to treatment. In these studies, we compared parameters (mutational load—NSEM, CD8 infiltration, and T cell clonality) in responding versus progressing lesions. These comparisons revealed statistically-significant differences in CD8^+^ T cell infiltrate between responding and progressing lesions (*p* = 0.009), but not NSEM and TCR clonality (Supplementary Fig. S18A), confirming that metastases with an increased CD8 T cell infiltrate present enhanced responses to both targeted therapy and immune checkpoint blockade. We next dichotomized therapeutic responses within patients and genomic, and immune markers were also compared between best responding and worst responding synchronous lesions, demonstrating higher levels of CD8, CD45RO, and PD-1 in responding lesions, though sample size was limited and this did not attain statistical significance (Supplementary Fig. S18B).

Based on these data, we next performed hierarchical clustering to assess the relative extent of genomic and immune heterogeneity in differential responses to targeted therapy and immune checkpoint blockade in synchronous melanoma metastases. In these studies, we found that though the majority of synchronous metastases clustered together genomically (Fig. 4d), few clustered together immunologically (Fig. 4e), suggesting that immune heterogeneity is more pervasive than genomic heterogeneity.

To quantify the degree of genomic and immune heterogeneity, we measured the average branch length required to cluster samples from the same patient, with a shorter branch length suggesting less heterogeneity. When we quantified the branch length based on genomic data in multiple metastases in a given patient and multiple regions within a given metastasis, we found both branch lengths to be comparable (Fig. 4f). However when we evaluated immune heterogeneity, we saw that the heterogeneity observed across metastases was significant, much more than would be expected across different regions of the same metastasis (Fig. 4f).

We next assessed this in therapeutic context and noted that genomic heterogeneity was consistent across treatment types (Fig. 4g). However, immune heterogeneity was far more pervasive, particularly in treatment-naïve or targeted therapy patients. Interestingly, immune heterogeneity was markedly lower in patients on immune checkpoint blockade, though statistical significance was not attained due to sample size, suggesting that treatment with immunotherapy may shape the immune system through selective pressure across metastases (Fig. 4g).

Following this, we sought to determine the relationship of the observed tumor heterogeneity with therapeutic response via radiomic analysis. To do this, we compared baseline radiomic indices derived from each metastatic lesion and plotted these against the radiologic response to treatment. In these studies, we observed positive correlations between baseline lesion entropy, homogeneity and eventual therapeutic response (*r* = 0.5919, *p* = 0.0123 and *r* = −0.5352, *p* = 0.0268, respectively) (Fig. 4h), suggesting that more homogeneous (less complex) lesions are more likely to respond to therapy. This relationship was also studied within individual patients when responses were dichotomized, and demonstrated similar results with higher entropy, dissimilarity and contrast in worst responding and higher energy and homogeneity in best responding synchronous lesions within each patient (Fig. 4i), suggesting that texture analysis at baseline may predict differential responses of synchronous metastases within a given patient through minimally invasive means. Of note, random forest analysis and hierarchical clustering of features from texture analysis, genomic, and immune correlates revealed that the texture features correlated with each other, and had a relatively higher importance in predicting response compared to genomic and immune correlates (Supplementary Fig. S19).

## Discussion

Unprecedented advances have been made in cancer treatment through the use of targeted therapy and immune checkpoint blockade,^1–4^ though a majority of patients do not derive durable clinical benefit. Melanoma is a prime example with growing evidence for the role of tumor heterogeneity in cancer development^7^ and resistance to therapy.^5^ Tremendous insight has been gained through the use of tumor sampling during the course of therapy.^6^ However, the standard approach is to sample only one lesion—often by core biopsy with a limited sample obtained—and may not be representative of the entire tumor or other metastatic lesions within the same patient.

Our understanding of the role of genomic heterogeneity is growing, with evidence for genomic heterogeneity observed within individual tumors,^9^ between synchronous metastases,^5, 7, 9^ and between primary and metastatic lesions.^7, 9^ The relationship of genomic heterogeneity to therapeutic responses to molecularly-targeted therapy has also been studied,^5^ though the role of genomic heterogeneity in the context of treatment with immune checkpoint blockade is not well understood. In addition to this, the role of immune heterogeneity is less defined.^10^

The data presented herein build on these prior studies, highlighting intrapatient heterogeneity of clinical responses not only to targeted therapy as described by others,^5^ but also to immune checkpoint blockade. Strikingly, heterogeneity of clinical responses was observed in the vast majority of patients on targeted therapy or immune checkpoint blockade (83%), suggesting that this is a prevalent phenomenon.

Importantly, the molecular and immune underpinnings behind the observed heterogeneity in clinical responses were comprehensively and deeply characterized in available tumor samples, yielding important insights into potential mechanisms of differential therapeutic responses that transcend those gained from prior studies.^5^ Though we did observe substantial intrapatient genomic heterogeneity in our studies, much greater overlap in mutational load and mutations in driver genes was observed across synchronous metastases than across the cohort, likely due to the seeding of synchronous metastases from a common primary tumor. Though not entirely explored in the context of melanoma, the overlap in mutational burden observed in our study between synchronous metastases is in line with previous reports in lung, kidney, and colon cancer.^9, 30, 33^ Furthermore, our patient cohort was largely reflective of the major driver mutations observed in cutaneous melanoma, with *BRAF* being detectable in 36% of patients, *NF1 in 14%* of patients, *NRAS* in 7% of patients, among others, though frequencies varied due to limited sample size.^23^ Furthermore, mutations in *BRAF* and *NRAS* were entirely conserved across synchronous metastases, in line with prior reports. Interestingly, mutations in *PPP6C*, *RAC1*, *SNX31*, *TACC1*, *STK19*, and *ARID2* were also detected in certain patients, with 64% of patients (9/14) harboring mutations in two or more driver genes. Importantly, the mutational landscape was consistent with cutaneous melanoma, with a dominant proportion of CT UV-associated mutations (79%, on average) found in all patients studied, and this signature being conserved across synchronous metastases.^22^

Immune heterogeneity, however, was far more pervasive, with divergent immune profiles and T cell repertoires in synchronous metastases of all patients studied (regardless of treatment category). Considering the HLA-restriction of these antigen-specific T cell responses, differences in the T cell repertoire across synchronous metastases may be related to HLA allele variants expressed by each patient, as well as variable expression levels of HLA molecules, even across synchronous metastases. Furthermore, these divergent immune profiles were associated with differential responses to therapy, more so than any genomic feature studied. However, it is also clear from these studies that genomic and immune factors are tightly related, as the mutational burden correlated positively with CD8^+^ T cell infiltrate and a more clonal T cell response.

These data have important clinical implications, as treatment decisions in patients with metastatic disease are often made based on mutational and immune analyses of a single tumor (and often from archival tissue of a primary lesion or distant metastasis). However, in light of our study and previously reported studies,^5^ biopsy of more than one lesion should be considered (when feasible) from patients with multiple tumors in order to obtain a better understanding of the spectrum of the disease, particularly in the setting of a mixed response to therapy. Furthermore, data acquired from multiple biopsies could be used to identify unifying molecular and immune features that could be targeted to achieve a more potent and durable response.

We also employed a novel radiomic approach to further delineate heterogeneity between lesions using texture analysis of conventional CT imaging evaluating several features known to reflect intratumoral heterogeneity. This approach was utilized to assess the likelihood of therapeutic response with provocative results, demonstrating that less complex lesions are more likely to respond to therapy, a phenomenon that has been observed in other tumor types.^42^ These pilot data are currently being investigated in a larger radiomic study though this is admittedly beyond the scope of this report. Nonetheless, comprehensive studies incorporating novel radiomic approaches may identify better (and less invasive) biomarkers of therapeutic response.

Despite these interesting results, several limitations exist in this study. Though clinical data were available on a large cohort of patients, tumor tissue from synchronous metastases was only available in a subset of patients for deep genomic and immune profiling. However, data obtained from this cohort were maximized through integrated genomic and immune analyses, and clearly illustrate the extent of genomic and immune heterogeneity observed in patients with synchronous melanoma metastases and how they relate to differential responses to therapy, even within this limited cohort. These results need to be validated in larger cohorts and also in other cancer types. In addition to this, the current study lacks incorporation of blood-based “liquid biopsies” due to a lack of available substrate, but efforts are underway to pair liquid biopsy approaches with tissue-based analysis in heterogeneity and other studies to assess for concordance. Furthermore, single-cell sequencing technologies are emerging and may provide a deeper understanding of both intralesional and interlesional heterogeneity though such studies were not incorporated into our analysis due to a lack of available substrate. Finally, though this study introduced some provocative findings of heterogeneity and response prediction using radiomics, this field is relatively nascent and the utility of such an approach still needs to be proven. However, efforts are underway to incorporate such analyses into larger cohorts of patients across multiple tumor types, and it is conceivable that such an approach could contribute significantly to predicting and assessing therapeutic responses. Taken together, studies such as these may ultimately inform us on the most effective choice and sequence of therapy in this age of personalized precision medicine.

## Methods

### Patient cohort

A cohort of 60 patients with synchronous melanoma metastases who had received first-line combination BRAF/MEK-targeted therapy (*n* = 30) or anti-PD-1 checkpoint blockade (*n* = 30) were studied for initial radiographic response assessment. For deeper molecular and immune profiling, a subset of patients with synchronous melanoma metastases resected were included (total of 15 patients - targeted therapy (*n* = 4), immune checkpoint blockade (*n* = 7), or treatment-naïve (*n* = 4)). Synchronous metastases were processed in parallel and molecular (WES, neoantigen prediction) and immune (flow cytometry, IHC, gene expression profiling, TCR sequencing) profiling was performed (Supplementary Fig. S20). All patients were treated at the University of Texas MD Anderson Cancer Center and had signed informed consent for collection and analysis of their tumor samples. Response was evaluated in synchronous metastases by measuring the best overall response within the first 6 months of treatment based on changes in tumor size from baseline in each lesion based on RECIST 1.1 using imaging available at the University of Texas MD Anderson Cancer Center.^20^ This protocol was approved by the Institutional Review Board.

### Radiomic assessment

Pre-treatment contrast-enhanced CT scans obtained on similar CT scanners using standardized imaging techniques were selected for analysis and loaded into the open-source 3D-Slicer software (www.slicer.org). The process of tumor segmentation by different software programs has been described extensively, with lesions being isolated and segmented through manual, automatic, or semiautomatic methods.^15^ Segmentation of synchronous melanoma metastases was performed by a board-certified radiologist with experience in tumor segmentation and texture analysis (Supplementary Fig. S21). As prior work has demonstrated that semi-automatic segmentation methods outperform manual segmentations in terms of repeatability, this method was utilized.^43^ The data obtained from segmentation were then imported into an in-house quantitative image software platform (Imaging Biomarker Explorer, IBEX), which was designed by using a commercial software package (Matlab, version 8.1.0; MathWorks, Natick, Mass)^44^ and extracts quantitative image features from delineated regions of interest. Specific second-order statistical descriptors were then obtained. These second-order statistical descriptors are defined as texture features representing statistical interrelationships between voxels with similar (or dissimilar) contrast values, representing intralesional heterogeneity.^15, 45, 46^ Specific texture features are based on different parent matrices capturing and describing the spatial intensity distribution.^43^ The gray level co-occurrence matrix counts voxel pairs with certain gray values at a predefined direction and distance from each other, and generates values reflective of heterogeneity such as entropy (randomness of the matrix), energy (orderliness), homogeneity (uniformity), dissimilarity (measurement of how different each element in the matrix is), and contrast (measurement of variation).^45^ These features were obtained and correlated with dichotomized intrapatient response based on RECIST 1.1 response criteria.^20^

### Whole exome sequencing

DNA was extracted from 33 fresh-frozen tumor samples following pathological assessment and confirmation of tumor content. Patient-matched and time-matched peripheral blood leukocytes were used as germline DNA control. Exome capture was performed on 500 ng of genomic DNA per sample based on KAPA library prep (Kapa Biosystems) using the Agilent SureSelect Human All Exon V4 kit according to the manufacturer’s instructions and paired-end multiplex sequencing of samples was performed on the Illumina HiSeq 2000 sequencing platform. The average sequencing depth was 179× with 76 BP read length per tumor sample (ranging from 97× to 314×, standard deviation ± 60).

Paired-end reads in FASTQ format were generated from BCL raw data off the sequencer by the Illumina CASAVA software. The reads were aligned to the reference human genome (UCSC Genome Browser, Hg19) using BWA by allowing a minimal seed length of 40, maximal edit distance of three bases. The alignments were then processed with the GATK software Best Practices of duplicate removal, indel re-alignment, and base re-calibration. Somatic SNVs (single-nucleotide variants) were called using the MuTect software on default settings along with post-calling filters: (a) total read count in tumor sample ≥ 20, (b) total read count in matched normal sample ≥ 10, (c) VAF (variant allele frequency) in tumor sample ≥ 0.01, (d) VAF in matched normal sample ≥ 0.01, and (e) SNVs in the databases of dbSNP129, 1000 Human Genomes, and ESP-6500 were removed.

WES data are available in the NCBI Sequence Read Archive public repository (BioProject ID PRJNA316754).

### NanoString analysis

Nanostring analysis was performed on tumor samples with sufficient frozen tissue available using a custom-designed 795-gene codeset including immune pathway genes and cancer pathway genes. Hematoxylin-stained and eosin-stained sections were prepared to evaluate tumor cellularity. Total RNA was extracted from each sample individually using RNeasy Mini Kit (QIAGEN). For each NanoString assay, 1 μg of total tissue RNA was isolated, mixed with a NanoString codeset mix and incubated at 65 °C overnight (16–18 h). The reaction mixes were loaded onto the NanoString nCounter Prep Station for binding and washing, and the resulting cartridge was transferred to the NanoString nCounter Digital Analyzer for scanning and data collection. A total of 600 fields were captured per sample to generate the raw digital counts for each sample.

Immune scores were calculated as the geometric mean of gene expression in cytolytic markers (*GZMA, GZMB, PRF1, GNLY*), HLA molecules (*HLA-A, HLA-B, HLA-C, HLA-E, HLA-F, HLA-G, HLA-H, HLA-DMA, HLA-DMB, HLA-DOA, HLA-DOB, HLA-DPA1, HLA-DPB1, HLA-DQA1, HLA-DQA2, HLA-DQB1, HLA-DRA, HLA-DRB1*), IFN-γ pathway genes (*IFNG, IFNGR1, IFNGR2, IRF1, STAT1, PSMB9*), chemokines (*CCR5, CCL3, CCL4, CCL5, CXCL9, CXCL10, CXCL11*), and adhesion molecules (*ICAM1, ICAM2, ICAM3, ICAM4, ICAM5, VCAM1*).

### Flow cytometry

Fresh melanoma tumors were cut into small pieces and digested for 1 h at 37 °C with collagenase A and DNAse I (both from Roche, Basel, Switzerland) under 225 RPM rotation. Following digestion, tumor suspension was filtered through a 70 μm cell strainer (BD Biosciences, Franklin Lakes, NJ), and single cell suspension was counted and plated into a 96-well round bottom plate for staining as previously described.^37^ In short, flow cytometry staining was carried out on five distinct CD45^+^ panels after LIVE/DEAD fixable aqua dead cell stain (Life Technologies, Carlsbad, CA) to look at CD4^+^ (CD3ε^+^CD4^+^) or CD8^+^ (CD3ε^+^CD8^+^) T lymphocytes, T regulatory (CD3ε^+^CD4^+^FoxP3^+^), macrophages (CD14^hi^CD11b^+^HLADR^+^), natural killer (NK- CD56^+^NKG2D^+^), γδ T (CD3ε^+^γδTCR^+^), B cells (CD3ε^−^CD19/20^+^HLA-DR^+^), myeloid dendritic cells (CD11c^+^HLA-DR^+^CD14^lo/−^), basophils (FCεR1α^+^CD117^−^CD11b^−^CD49d^+^), mast cells (FCεR1a^+^CD117^+^CD11b^−^CD49d^+^), neutrophils (CD15^+^CD11b^+^CD49d^−^), and eosinophils (CD15^+^CD11b^+^CD49d^+^). Following staining of surface markers, cells were fixed with Cytofix/Cytoperm concentrate (eBioscience, San Diego, CA) for intracellular staining and acquired on a BD LSRFortessa II flow cytometer (BD Biosciences). All data analysis was performed with FlowJo version 10 (Tree Star Inc., Ashland, OR).

### Immunohistochemistry

From each formalin-fixed paraffin-embedded tissue block, a hematoxylin and eosin-stained slide was examined by a pathologist to confirm the presence of tumor. Four microns-thick sections were cut from a representative tumor block selected from each case for IHC analysis. Heavily pigmented samples underwent melanin bleaching with potassium permanganate and oxalic acid prior to CD8 staining. IHC was performed using a Leica Bond Max automated stainer (Leica Biosystems, Buffalo Grove, IL). The primary antibodies employed included programmed death-ligand 1 (PD-L1) clone E1L3N (1:100, Cell Signaling Technology, Beverly, MA), PD-1 clone EPR4877(2) (1:250, Epitomics, Cambridge, MA), CD3 polyclonal (1:100, DAKO, Carpinteria, CA), CD4 clone 4B12 (1:80, Leica Biosystems), CD8 clone C8/144B (1:25, Thermo Scientific, Waltham, MA), CD45RO clone UCHL1 (ready to use, Leica Biosystems), CD57 clone HNK-1 (1:40, BD Biosciences), CD68 clone PG-M1 (1:450, DAKO), FoxP3 clone 206D (1:50, BioLegend), Granzyme B clone 11F1 (ready to use, Leica Microsystems) and OX40 clone ACT-35 (1:100, eBioscience). All slides were stained using previously optimized conditions including a positive control (human placenta for PD-L1 and human tonsil for other markers) and a non-primary antibody control. The IHC reaction was detected using Leica Bond Polymer Refine detection kit (Leica Biosystems) and diaminobenzidine was used as chromogen. Counterstaining was done using Hematoxylin. All IHC-stained slides were converted into high-resolution digital images of the whole tissue (e-slide) using a pathology scanner (Aperio AT Turbo, Leica Biosystems). The e-slides were then analyzed using the Aperio Image Toolbox analysis software (Leica Biosystems). From each e-slide, five random 1-mm^2^ areas within the tumor region were chosen by a pathologist for digital analysis. PD-L1 expression was evaluated in tumor cells using an *H*-score, which includes the percentage of positive cells showing a membrane staining pattern (0 to 100) and intensity of the staining (0 to 3+), with a total score ranging from 0 to 300. All other markers were evaluated as density of cells, defined as the number of positive cells per area (1 mm^2^) regardless of the intensity. The final score for each marker was expressed as the average score of the five areas analyzed within the tumor region. The final scores for each marker from each patient were then transferred to a database for statistical analysis.^47^

### TCR sequencing

CDR3 regions were sequenced by ImmunoSeq^TM^ sequencing (Adaptive Biotechnologies), with primers annealing to V and J segments, resulting in amplification of rearranged VDJ segments from each cell. Clonality values were obtained through the ImmunoSeq Analyzer software and measured as 1-(entropy)/log2(# of productive unique sequences), with entropy taking into account clone frequency.

### Neoantigen prediction

WES data were reviewed for NSEM and all possible 8-mer to 12-mer peptides spanning NSEM were generated, and compared with wild type peptides. Binding affinity was evaluated taking into account patient HLA with the NetMHC 3.4 algorithm. Candidate peptides were considered HLA binders when IC50 < 500 nM. High-affinity HLA binders were defined as peptides predicted to have an IC50 < 100 nM. Neoantigen plots were generated with Tableau software (Seattle, WA).

### Statistical analysis

Statistical analyses were performed using GraphPad Prism software and R statistical packages. Immune variables were logarithm-transformed in the downstream analysis.

#### Sample clustering

Agglomerative Ward’s linkage hierarchical clustering analysis of the samples was conducted using genomic and immune variables separately. For genomic variables, which had zero values, Euclidean distance was used as the distance measurement. For continuous immune variables, Pearson correlation was used as the distance measurement. Hierarchical clustering plots are drawn with the sample ID and therapy label attached. Median branch lengths were compared between groups using the Wilcoxon rank sum test. All statistical analyses were two-sided at an alpha level of 0.05. R (version: 3.2.3) with package “ClassComparison” and “ClassDiscovery” and STATA 14 (College Station, TX) used to conduct all analyses and generate plots.

#### Tumor size change versus mutation/genomic/immune correlations

The association between measurements of markers (mutation, immune, or genomic variables) and response status of tumor size change was assessed using an analysis of variance (ANOVA) model. We considered two binary response variables: (1) within a patient, the sample having the best response corresponding to the largest tumor size change was indexed by 1; and 0 otherwise; (2) within a patient, the sample having the worst response corresponding to the smallest tumor size change was indexed by 1; and 0 otherwise. The ANOVA model was used to explore the associations between each marker and each of the two response variables, with a random intercept incorporated to account for correlations among the samples from the same tumor location in one patient. Analysis was performed on all patients with response data. Multiple testing was controlled for using false discovery rate with the Benjamini–Hochberg procedure. NSEM, CD8 and clonality were dichotomized at the median and compared to response based on RECIST1.1 using the Fischer’s exact test.

The “randomForest” R package version 4.6–12 was implemented for random forest analysis. The following parameters were used: 1000 trees, node size of 1, and mtry values equal to the square root of the total number of variables. The Gini index was used as a splitting rule. Variable importance scores were calculated as the total decrease in node impurities from splitting on the variable, averaged over 1000 trees. All samples were used as training set and out-of-bag classification error rate was calculated as an estimate of test error. Hierarchical clustering between variables was performed on 11 variables across 14 samples. Ward linkage method (method = ward.D2 in hclust function) was used with a Spearman correlation metric distance by heatmap. *R* function and variable values were scaled by rows (*z*-score).

## Electronic supplementary material

Supplementary Figure Legends

Supplemental Figures S1-S21

Supplemental Table Legends

Supplemental Tables S1-S7
