# Supplementary material for: Genomic and immune heterogeneity are associated with differential responses to therapy in melanoma
Source: NPJ Genom Med. 2017 Apr 7;2:10. doi: 10.1038/s41525-017-0013-8 (PMC5557036; doi:10.1038/s41525-017-0013-8)
Supplement: Supplementary file 1 — Supplementary Figure Legends [file 41525_2017_13_MOESM1_ESM.docx]

**Supplementary Figure Legends**

**Figure S1. Tumor sites, treatment history, genomic and immune data in patient #1. A)** Schematic outlining sites of metastases, as well as treatment history. Computerized axial tomography (CAT) scans of two synchronous metastases from a patient on targeted therapy at baseline and 12 weeks later. Red circles highlight tumor sites. Percentages indicate change in tumor size from baseline to 12 weeks as measured by RECIST. **B)** Shown is the overlap in NSEM between both metastases within this patient, and the mutational signature in each metastasis (**C**) as evaluated by whole exome sequencing (WES). **D**) Predicted neoantigens are plotted based on IC50, with blue and red dots representing tumor-restricted neoantigen candidates and grey dots those shared. **E)** Stacked bars showing the relative abundance of immune cell subsets. Results are shown as a percentage of CD45+ immune cells within each metastasis. **F**) Immunohistochemical staining of formalin-fixed paraffin-embedded tissue for PD-L1, PD-1, CD3, CD4, CD8, CD57, CD45RO, GzmB, FoxP3, CD68, and OX40 in both metastases within patient. Values are represented as Counts/mm^2^ or H-Score (PD-L1). Unavailable sample due to pigmentation. **G**) Frequency of each TCR clone detected in both metastases within patient as evaluated using CDR3 sequencing. **H**) Hive plot representation of NSEM, CD8% and TCR clonality as normalized within patient.

**Figure S2. Tumor sites, treatment history, genomic and immune data in patient #2. A)** Schematic outlining sites of metastases, as well as treatment history. Computerized axial tomography (CAT) scans of two synchronous metastases from a patient on targeted therapy at baseline and 6 weeks later. Red circles highlight tumor sites. Percentages indicate change in tumor size from baseline to 6 weeks as measured by RECIST. **B)** Shown is the overlap in NSEM between both metastases within this patient, and the mutational signature in each metastasis (**C**) as evaluated by whole exome sequencing (WES). **D**) Predicted neoantigens are plotted based on IC50, with blue and red dots representing tumor-restricted neoantigen candidates and grey dots those shared. **E)** Stacked bars showing the relative abundance of immune cell subsets. Results are shown as a percentage of CD45+ immune cells within each metastasis. **F**) Immunohistochemical staining of formalin-fixed paraffin-embedded tissue for PD-L1, PD-1, CD3, CD4, CD8, CD57, CD45RO, GzmB, FoxP3, CD68, and OX40 in both metastases within patient. Values are represented as Counts/mm^2^ or H-Score (PD-L1). Unavailable sample due to pigmentation. **G**) Frequency of each TCR clone detected in both metastases within patient as evaluated using CDR3 sequencing. **H**) Hive plot representation of NSEM, CD8% and TCR clonality as normalized within patient.

**Figure S3. Tumor sites, treatment history, genomic and immune data in patient #3. A)** Schematic outlining sites of metastases, as well as treatment history. **B)** Shown is the overlap in NSEM between both metastases within this patient, and the mutational signature in each metastasis (**C**) as evaluated by whole exome sequencing (WES). **D**) Predicted neoantigens are plotted based on IC50, with blue and red dots representing tumor-restricted neoantigen candidates and grey dots those shared. **E)** Stacked bars showing the relative abundance of immune cell subsets. Results are shown as a percentage of CD45+ immune cells within each metastasis. **F**) Immunohistochemical staining of formalin-fixed paraffin-embedded tissue for PD-L1, PD-1, CD3, CD4, CD8, CD57, CD45RO, GzmB, FoxP3, CD68, and OX40 in both metastases within patient. Values are represented as Counts/mm^2^ or H-Score (PD-L1). Unavailable sample due to pigmentation. **G**) Frequency of each TCR clone detected in both metastases within patient as evaluated using CDR3 sequencing. **H**) Hive plot representation of NSEM, CD8% and TCR clonality as normalized within patient.

**Figure S4. Tumor sites, treatment history, genomic and immune data in patient #4. A)** Schematic outlining sites of metastases, as well as treatment history. Computerized axial tomography (CAT) scans of three synchronous metastases from a patient on targeted therapy at baseline and 25 weeks later. Red circles highlight tumor sites. Percentages indicate change in tumor size from baseline to 25 weeks as measured by RECIST. **B)** Shown is the overlap in NSEM between both metastases within this patient, and the mutational signature in each metastasis (**C**) as evaluated by whole exome sequencing (WES). **D**) Predicted neoantigens are plotted based on IC50, with blue and red dots representing tumor-restricted neoantigen candidates and grey dots those shared. **E)** Stacked bars showing the relative abundance of immune cell subsets. Results are shown as a percentage of CD45+ immune cells within each metastasis. **F**) Immunohistochemical staining of formalin-fixed paraffin-embedded tissue for PD-L1, PD-1, CD3, CD4, CD8, CD57, CD45RO, GzmB, FoxP3, CD68, and OX40 in both metastases within patient. Values are represented as Counts/mm^2^ or H-Score (PD-L1). Unavailable markers due to sample pigmentation. **G**) Frequency of each TCR clone detected in both metastases within patient as evaluated using CDR3 sequencing. **H**) Hive plot representation of NSEM, CD8% and TCR clonality as normalized within patient. Metastasis 3 was excluded due to high necrosis.

**Figure S5. Tumor sites, treatment history, genomic and immune data in patient #5. A)** Schematic outlining sites of metastases, as well as treatment history. Computerized axial tomography (CAT) scans of two synchronous metastases from a patient on targeted therapy at baseline and 11 weeks later. Red circles highlight tumor sites. Percentages indicate change in tumor size from baseline to 11 weeks as measured by RECIST. **B)** Shown is the overlap in NSEM between both metastases within this patient, and the mutational signature in each metastasis (**C**) as evaluated by whole exome sequencing (WES). **D**) Predicted neoantigens are plotted based on IC50, with blue and red dots representing tumor-restricted neoantigen candidates and grey dots those shared. **E)** Stacked bars showing the relative abundance of immune cell subsets. Results are shown as a percentage of CD45+ immune cells within each metastasis. **F**) Immunohistochemical staining of formalin-fixed paraffin-embedded tissue for PD-L1, PD-1, CD3, CD4, CD8, CD57, CD45RO, GzmB, FoxP3, CD68, and OX40 in both metastases within patient. Values are represented as Counts/mm^2^ or H-Score (PD-L1). Unavailable markers due to sample pigmentation. **G**) Frequency of each TCR clone detected in both metastases within patient as evaluated using CDR3 sequencing. **H**) Hive plot representation of NSEM, CD8% and TCR clonality as normalized within patient.

**Figure S6. Tumor sites, treatment history, genomic and immune data in patient #6. A)** Schematic outlining sites of metastases, as well as treatment history. **B)** Shown is the overlap in NSEM between both metastases within this patient, and the mutational signature in each metastasis (**C**) as evaluated by whole exome sequencing (WES). **D**) Predicted neoantigens are plotted based on IC50, with blue and red dots representing tumor-restricted neoantigen candidates and grey dots those shared. **E)** Stacked bars showing the relative abundance of immune cell subsets. Results are shown as a percentage of CD45+ immune cells within each metastasis. **F**) Immunohistochemical staining of formalin-fixed paraffin-embedded tissue for PD-L1, PD-1, CD3, CD4, CD8, CD57, CD45RO, GzmB, FoxP3, CD68, and OX40 in both metastases within patient. Values are represented as Counts/mm^2^ or H-Score (PD-L1). **G**) Frequency of each TCR clone detected in both metastases within patient as evaluated using CDR3 sequencing. **H**) Hive plot representation of NSEM, CD8% and TCR clonality as normalized within patient.

**Figure S7. Tumor sites, treatment history, genomic and immune data in patient #7. A)** Schematic outlining sites of metastases, as well as treatment history. **B)** Shown is the overlap in NSEM between both metastases within this patient, and the mutational signature in each metastasis (**C**) as evaluated by whole exome sequencing (WES). **D**) Predicted neoantigens are plotted based on IC50, with blue and red dots representing tumor-restricted neoantigen candidates and grey dots those shared. **E)** Stacked bars showing the relative abundance of immune cell subsets. Results are shown as a percentage of CD45+ immune cells within each metastasis. **F**) Immunohistochemical staining of formalin-fixed paraffin-embedded tissue for PD-L1, PD-1, CD3, CD4, CD8, CD57, CD45RO, GzmB, FoxP3, CD68, and OX40 in both metastases within patient. Values are represented as Counts/mm^2^ or H-Score (PD-L1). **G**) Frequency of each TCR clone detected in both metastases within patient as evaluated using CDR3 sequencing. **H**) Hive plot representation of NSEM, CD8% and TCR clonality as normalized within patient.

**Figure S8. Tumor sites, treatment history, genomic and immune data in patient #8. A)** Schematic outlining sites of metastases, as well as treatment history. **B)** Shown is the overlap in NSEM between metastases within this patient, and the mutational signature in each metastasis (**C**) as evaluated by whole exome sequencing (WES). **D**) Predicted neoantigens are plotted based on IC50, with colored dots representing tumor-restricted neoantigen candidates and grey dots those shared. **E)** Stacked bars showing the relative abundance of immune cell subsets. Results are shown as a percentage of CD45+ immune cells within each metastasis. **F**) Immunohistochemical staining of formalin-fixed paraffin-embedded tissue for PD-L1, PD-1, CD3, CD4, CD8, CD57, CD45RO, GzmB, FoxP3, CD68, and OX40 in both metastases within patient. Values are represented as Counts/mm^2^ or H-Score (PD-L1). **G**) Frequency of each TCR clone detected in both metastases within patient as evaluated using CDR3 sequencing. **H**) Hive plot representation of NSEM, CD8% and TCR clonality as normalized within patient.

**Figure S9. Tumor sites, treatment history, genomic and immune data in patient #9. A)** Schematic outlining sites of metastases, as well as treatment history. Computerized axial tomography (CAT) scans of four synchronous metastases from a patient on targeted therapy at baseline and 15 weeks later. Red circles highlight tumor sites. Percentages indicate change in tumor size from baseline to 15 weeks as measured by RECIST. **B)** Shown is the overlap in NSEM between metastases within this patient, and the mutational signature in each metastasis (**C**) as evaluated by whole exome sequencing (WES). **D**) Predicted neoantigens are plotted based on IC50, with colored dots representing tumor-restricted neoantigen candidates and grey dots those shared. **E)** Stacked bars showing the relative abundance of immune cell subsets. Results are shown as a percentage of CD45+ immune cells within each metastasis. **F**) Immunohistochemical staining of formalin-fixed paraffin-embedded tissue for PD-L1, PD-1, CD3, CD4, CD8, CD57, CD45RO, GzmB, FoxP3, CD68, and OX40 in both metastases within patient. Values are represented as Counts/mm^2^ or H-Score (PD-L1). Unavailable samples due pigmentation. **G**) Frequency of each TCR clone detected in both metastases within patient as evaluated using CDR3 sequencing. **H**) Hive plot representation of NSEM, CD8% and TCR clonality as normalized within patient. Metastasis 3 was excluded due to high necrosis.

**Figure S10. Tumor sites, treatment history, genomic and immune data in patient #10. A)** Schematic outlining sites of metastases, as well as treatment history. Computerized axial tomography (CAT) scans of two synchronous metastases from a patient on targeted therapy at baseline and 11 weeks later. Red circles highlight tumor sites. Percentages indicate change in tumor size from baseline to 11 weeks as measured by RECIST. **B)** Shown is the overlap in NSEM between both metastases within this patient, and the mutational signature in each metastasis (**C**) as evaluated by whole exome sequencing (WES). **D**) Predicted neoantigens are plotted based on IC50, with colored dots representing tumor-restricted neoantigen candidates and grey dots those shared. **E)** Stacked bars showing the relative abundance of immune cell subsets. Results are shown as a percentage of CD45+ immune cells within each metastasis. **F**) Immunohistochemical staining of formalin-fixed paraffin-embedded tissue for PD-L1, PD-1, CD3, CD4, CD8, CD57, CD45RO, GzmB, FoxP3, CD68, and OX40 in both metastases within patient. Values are represented as Counts/mm^2^ or H-Score (PD-L1). Unavailable samples and markers due to sample pigmentation. **G**) Frequency of each TCR clone detected in both metastases within patient as evaluated using CDR3 sequencing. **H**) Hive plot representation of NSEM, CD8% and TCR clonality as normalized within patient.

**Figure S11. Tumor sites, treatment history, genomic and immune data in patient #11. A)** Schematic outlining sites of metastases, as well as treatment history. **B)** Shown is the overlap in NSEM between both metastases within this patient, and the mutational signature in each metastasis (**C**) as evaluated by whole exome sequencing (WES). **D**) Predicted neoantigens are plotted based on IC50, with blue and red dots representing tumor-restricted neoantigen candidates and grey dots those shared. **E)** Stacked bars showing the relative abundance of immune cell subsets. Results are shown as a percentage of CD45+ immune cells within each metastasis. **F**) Immunohistochemical staining of formalin-fixed paraffin-embedded tissue for PD-L1, PD-1, CD3, CD4, CD8, CD57, CD45RO, GzmB, FoxP3, CD68, and OX40 in both metastases within patient. Values are represented as Counts/mm^2^ or H-Score (PD-L1). **G**) Frequency of each TCR clone detected in both metastases within patient as evaluated using CDR3 sequencing. **H**) Hive plot representation of NSEM, CD8% and TCR clonality as normalized within patient.

**Figure S12. Tumor sites, treatment history, genomic and immune data in patient #12. A)** Schematic outlining sites of metastases, as well as treatment history. **B)** Shown is the overlap in NSEM between both metastases within this patient, and the mutational signature in each metastasis (**C**) as evaluated by whole exome sequencing (WES). **D**) Predicted neoantigens are plotted based on IC50, with blue and red dots representing tumor-restricted neoantigen candidates and grey dots those shared. **E)** Stacked bars showing the relative abundance of immune cell subsets. Results are shown as a percentage of CD45+ immune cells within each metastasis. **F**) Immunohistochemical staining of formalin-fixed paraffin-embedded tissue for PD-L1, PD-1, CD3, CD4, CD8, CD57, CD45RO, GzmB, FoxP3, CD68, and OX40 in both metastases within patient. Values are represented as Counts/mm^2^ or H-Score (PD-L1). **G**) Frequency of each TCR clone detected in both metastases within patient as evaluated using CDR3 sequencing. **H**) Hive plot representation of NSEM, CD8% and TCR clonality as normalized within patient.

**Figure S13. Tumor sites, treatment history, genomic and immune data in patient #13. A)** Schematic outlining sites of metastases, as well as treatment history. **B)** Shown is the overlap in NSEM between both metastases within this patient, and the mutational signature in each metastasis (**C**) as evaluated by whole exome sequencing (WES). **D**) Predicted neoantigens are plotted based on IC50, with blue and red dots representing tumor-restricted neoantigen candidates and grey dots those shared. **E)** Stacked bars showing the relative abundance of immune cell subsets. Results are shown as a percentage of CD45+ immune cells within each metastasis. **F**) Immunohistochemical staining of formalin-fixed paraffin-embedded tissue for PD-L1, PD-1, CD3, CD4, CD8, CD57, CD45RO, GzmB, FoxP3, CD68, and OX40 in both metastases within patient. Values are represented as Counts/mm^2^ or H-Score (PD-L1). **G**) Frequency of each TCR clone detected in both metastases within patient as evaluated using CDR3 sequencing. **H**) Hive plot representation of NSEM, CD8% and TCR clonality as normalized within patient.

**Figure S14. Tumor sites, treatment history, genomic and immune data in patient #14. A)** Schematic outlining sites of metastases, as well as treatment history. Computerized axial tomography (CAT) scans of two synchronous metastases from a patient on targeted therapy at baseline and 7 weeks later. Red circles highlight tumor sites. Percentages indicate change in tumor size from baseline to 7 weeks as measured by RECIST. **B)** Shown is the overlap in NSEM between both metastases within this patient, and the mutational signature in each metastasis (**C**) as evaluated by whole exome sequencing (WES). **D**) Predicted neoantigens are plotted based on IC50, with blue and red dots representing tumor-restricted neoantigen candidates and grey dots. **E)** Stacked bars showing the relative abundance of immune cell subsets. Results are shown as a percentage of CD45+ immune cells within each metastasis. **F**) Immunohistochemical staining of formalin-fixed paraffin-embedded tissue for PD-L1, PD-1, CD3, CD4, CD8, CD57, CD45RO, GzmB, FoxP3, CD68, and OX40 in both metastases within patient. Values are represented as Counts/mm^2^ or H-Score (PD-L1). Unavailable samples and markers due to sample pigmentation. **G**) Frequency of each TCR clone detected in both metastases within patient as evaluated using CDR3 sequencing. **H**) Hive plot representation of NSEM, CD8% and TCR clonality as normalized within patient.

**Figure S15. Tumor sites, treatment history, genomic and immune data in patient #15. A)** Schematic outlining site of metastasis, as well as treatment history. **B)** Shown is the overlap in NSEM between regions from the metastasis, and the mutational signature in each sample (**C**) as evaluated by whole exome sequencing (WES). **D**) Predicted neoantigens in plotted based on IC50, with blue dots representing tumor-restricted neoantigen candidates and grey dots those shared. **E)** Stacked bars showing the relative abundance of immune cell subsets. Results are shown as a percentage of CD45+ immune cells within each metastasis. **F**) Immunohistochemical staining of formalin-fixed paraffin-embedded tissue for PD-L1, PD-1, CD3, CD4, CD8, CD57, CD45RO, GzmB, FoxP3, CD68, and OX40 in both metastases within patient. Values are represented as Counts/mm^2^ or H-Score (PD-L1). **G**) Frequency of each TCR clone detected in both metastases within patient as evaluated using CDR3 sequencing. **H**) Hive plot representation of NSEM, CD8% and TCR clonality as normalized within patient.

**Figure S16. Melanoma driver gene mutation allele frequency in synchronous melanoma metastases.** Mutational status and allelic frequency of melanoma driver genes following whole exome sequencing (WES).

**Figure S17. Limited correlation between rank of top 10 and top 100 clones across synchronous metastases.** Top 100 T cell clones in each patient based on their relative frequency in each synchronous metastasis. Shown are Spearman rank-correlations for the top 10 and top 100 clones in each patient. Green, Treatment-naïve; Red, Targeted therapy; Blue, Immunotherapy.

**Figure S18. Intrapatient genomic and immune correlations with tumor response. A)** The percentage of lesions with values above the median for NSEM, CD8% and TCR clonality in our patient cohort based on observed clinical benefit (SD, PR) or PD. PD = progressive disease, SD = stable disease. **B)** Correlation analysis of genomic (green, NSEM and neoantigen load) and immune (blue, flow cytometry; red, immunohistochemistry; purple, TCR sequencing) in relation to best and worst responding lesions within each patient with available clinical response data. Presented is the p-value for correlation of parameter with indicated relative response. Dotted line = 0.05

**Figure S19.** **Importance scores by random forest analysis and hierarchical clustering of variables by response status. A)** Importance scores for all variables available across all patients studied as calculated by random forest analysis. **B)** Importance scores for texture features (contrast), CD8, TCR clonality, neoantigens, and mutational load across all patients studied as calculated by random forest analysis. **C)** Hierarchical clustering from all variables available across all patients studied ordered by response status (Purple: Worst Response, Teal: Best Response). Ward linkage method was used with a Spearman correlation metric distance and variable values were scaled by rows (z-score). Green, Texture feature; Red, Genomic factor; Blue, Immune factor.

**Figure S20. Experimental plan.** Synchronous metastases were harvested, processed in parallel and molecular (whole exome sequencing, neoantigen expression) and immune (flow cytometry, immunohistochemistry, gene expression profiling, and TCR sequencing) profiling was performed.

**Figure S21. Example of lesion segmentation using 3D-Slicer. A)** Axial, sagittal and coronal views of a pre-treatment CT scan of the left lower extremity demonstrate a heterogeneous metastasis in the left popliteal fossa.  **B)** Same CT images show segmentation of the left popliteal fossa lesion (highlighted in green, bottom) and creation of a corresponding 3-dimentional model (top) in preparation for data exportation and texture analysis.
