# Supplementary material for: Genomic and immune heterogeneity are associated with differential responses to therapy in melanoma
Source: NPJ Genom Med. 2017 Apr 7;2:10. doi: 10.1038/s41525-017-0013-8 (PMC5557036; doi:10.1038/s41525-017-0013-8)

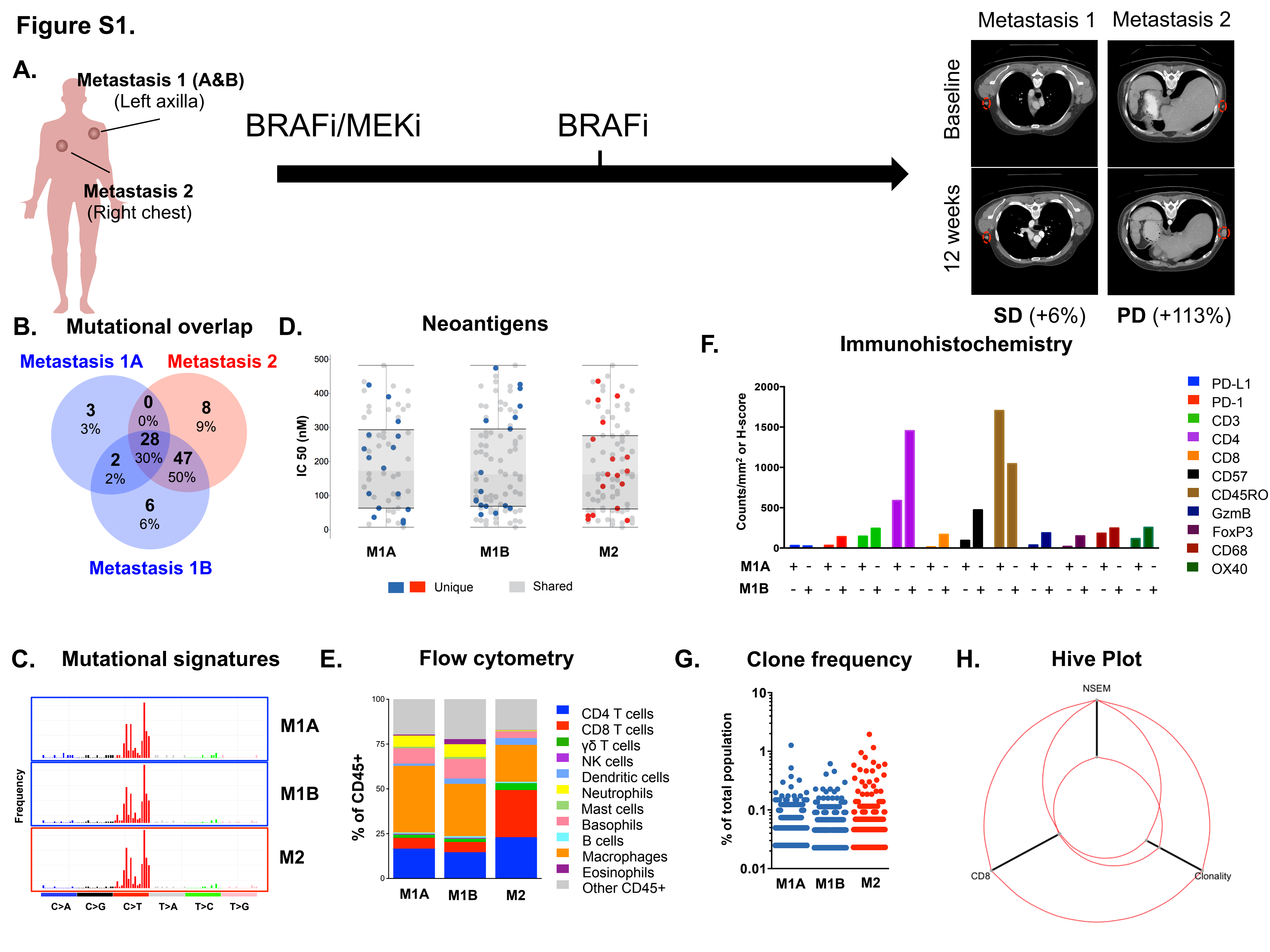

Figure S1.
A.
Metastasis 1 (A&B)
(Left axilla)
Metastasis 2
(Right chest)
BRAFi/MEKi
BRAFi
Metastasis 1
Metastasis 2
Baseline
12 weeks
SD (+6%)
PD (+113%)
B.
Mutational overlap
Metastasis 1A
Metastasis 2
Metastasis 1B
3
3%
0
0%
8
9%
28
30%
2
2%
47
50%
6
6%
D.
Neoantigens
IC 50 (nM)
M1A
M1B
M2
Unique
Shared
F.
Immunohistochemistry
Counts/mm² or H-score
PD-L1
PD-1
CD3
CD4
CD8
CD57
CD45RO
GzmB
FoxP3
CD68
OX40
M1A
M1B
C.
Mutational signatures
Frequency
M1A
M1B
M2
C>A
C>G
C>T
T>A
T>C
T>G
E.
Flow cytometry
% of CD45+
CD4 T cells
CD8 T cells
γδ T cells
NK cells
Dendritic cells
Neutrophils
Mast cells
Basophils
B cells
Macrophages
Eosinophils
Other CD45+
G.
Clone frequency
% of total population
H.
Hive Plot
NSEM
CD8
Clonality

Figure S2.

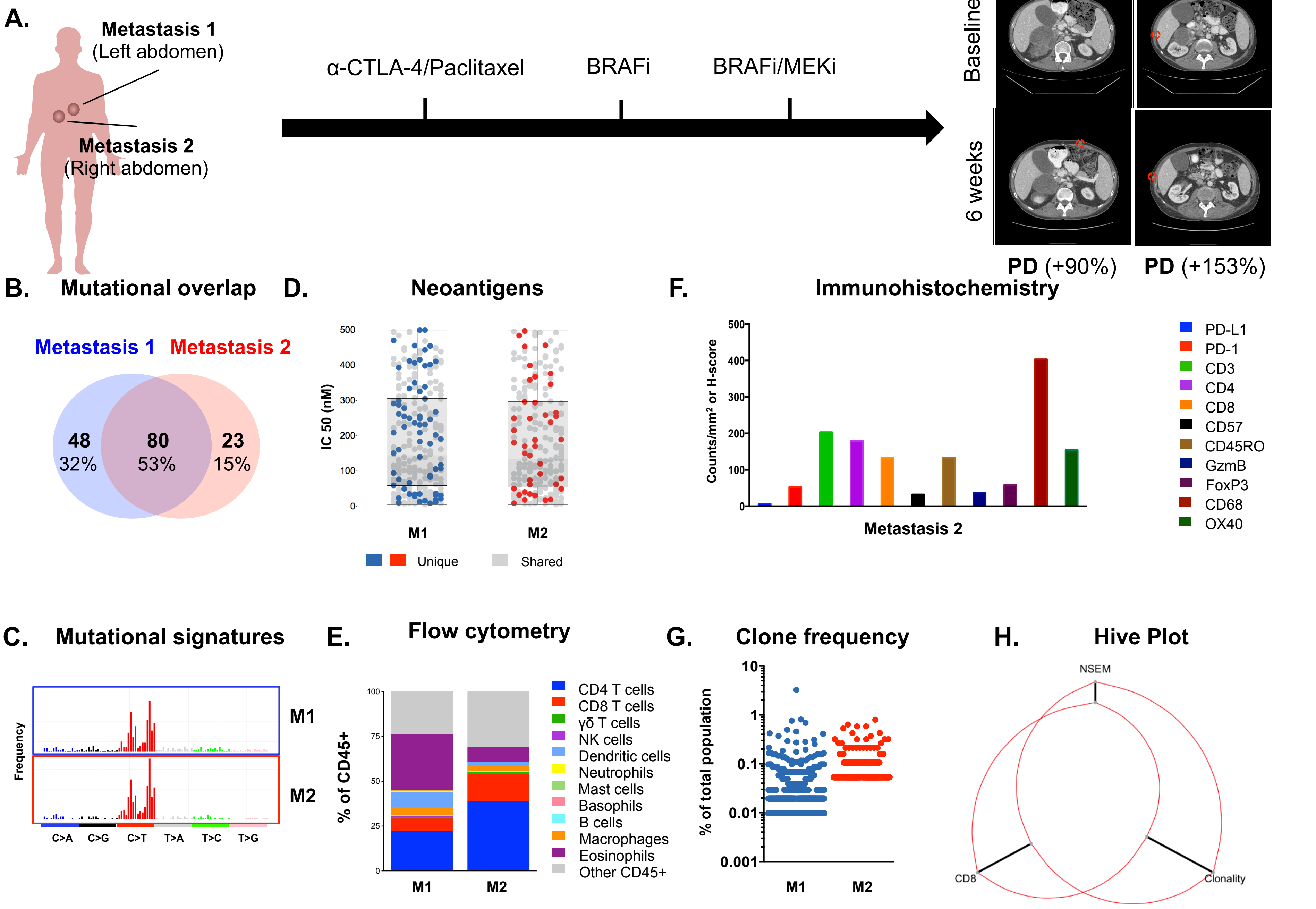

A.
Metastasis 1
(Left abdomen)
Metastasis 2
(Right abdomen)
α-CTLA-4/Paclitaxel
BRAFi
BRAFi/MEKi
Metastasis 1
Metastasis 2
Baseline
6 weeks
PD (+90%)
PD (+153%)
B.
Mutational overlap
Metastasis 1
Metastasis 2
48
32%
80
53%
23
15%
C.
Mutational signatures
Frequency
M1
M2
C>A
C>G
C>T
T>A
T>C
T>G
D.
Neoantigens
IC 50 (nM)
500
400
300
200
100
0
M1
M2
Unique
Shared
E.
Flow cytometry
% of CD45+
100
75
50
25
0
M1
M2
CD4 T cells
CD8 T cells
γδ T cells
NK cells
Dendritic cells
Neutrophils
Mast cells
Basophils
B cells
Macrophages
Eosinophils
Other CD45+
F.
Immunohistochemistry
Counts/mm² or H-score
500
400
300
200
100
0
Metastasis 2
PD-L1
PD-1
CD3
CD4
CD8
CD57
CD45RO
GzmB
FoxP3
CD68
OX40
G.
Clone frequency
% of total population
10
1
0.1
0.01
0.001
M1
M2
H.
Hive Plot
NSEM
CD8
Clonality

Figure S3.

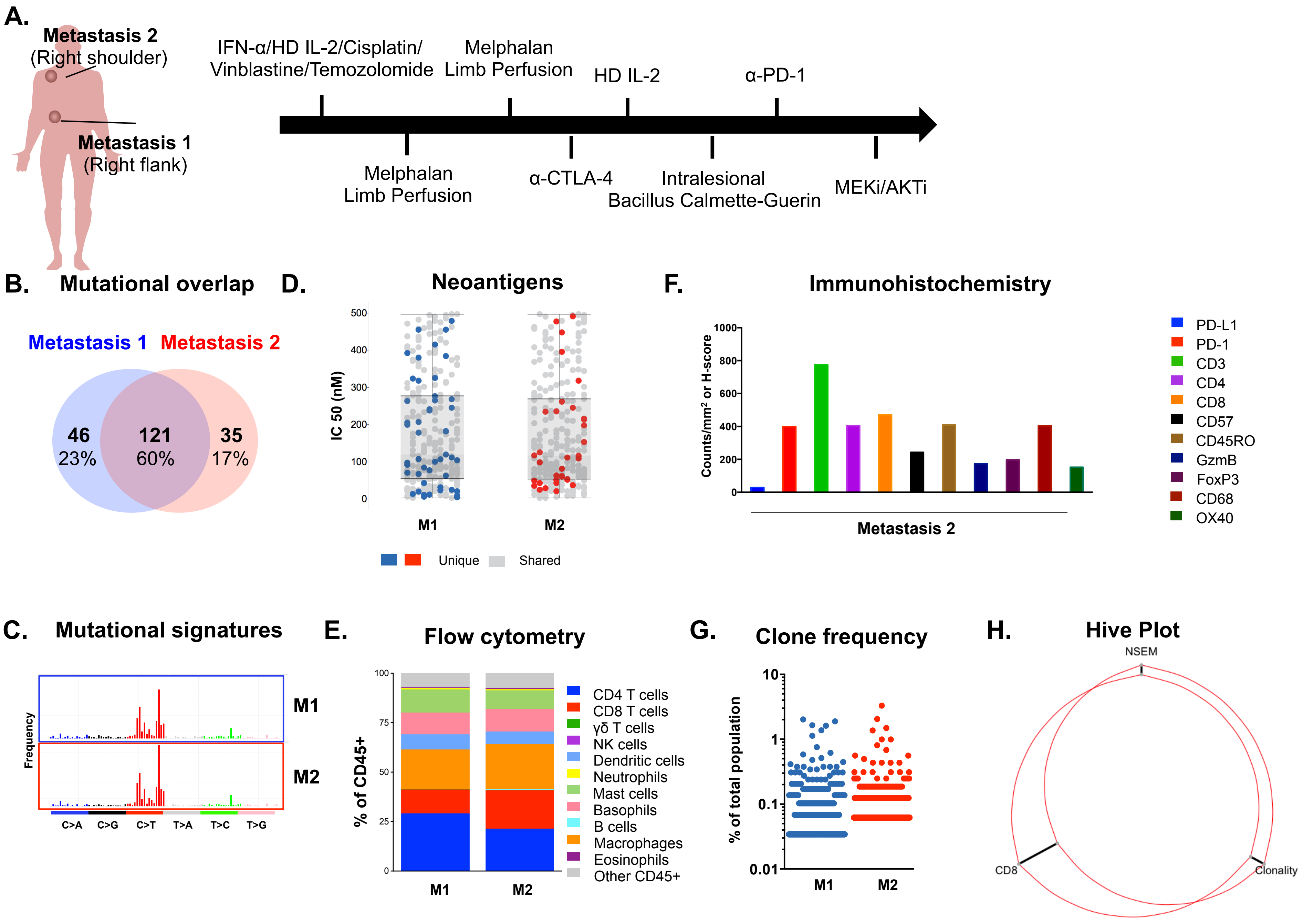
A.
Metastasis 2
(Right shoulder)
Metastasis 1
(Right flank)
IFN-α/HD IL-2/Cisplatin/
Vinblastine/Temozolomide
Melphalan
Limb Perfusion
HD IL-2
α-PD-1
Melphalan
Limb Perfusion
α-CTLA-4
Intralesional
Bacillus Calmette-Guerin
MEKi/AKTi
B.
Mutational overlap
Metastasis 1
Metastasis 2
46
23%
121
60%
35
17%
D.
Neoantigens
IC 50 (nM)
0
100
200
300
400
500
M1
M2
Unique
Shared
F.
Immunohistochemistry
Counts/mm² or H-score
0
200
400
600
800
1000
Metastasis 2
PD-L1
PD-1
CD3
CD4
CD8
CD57
CD45RO
GzmB
FoxP3
CD68
OX40
C.
Mutational signatures
Frequency
M1
M2
C>A
C>G
C>T
T>A
T>C
T>G
E.
Flow cytometry
% of CD45+
0
25
50
75
100
M1
M2
CD4 T cells
CD8 T cells
γδ T cells
NK cells
Dendritic cells
Neutrophils
Mast cells
Basophils
B cells
Macrophages
Eosinophils
Other CD45+
G.
Clone frequency
% of total population
0.01
0.1
1
10
M1
M2
H.
Hive Plot
NSEM
CD8
Clonality

Figure S4.

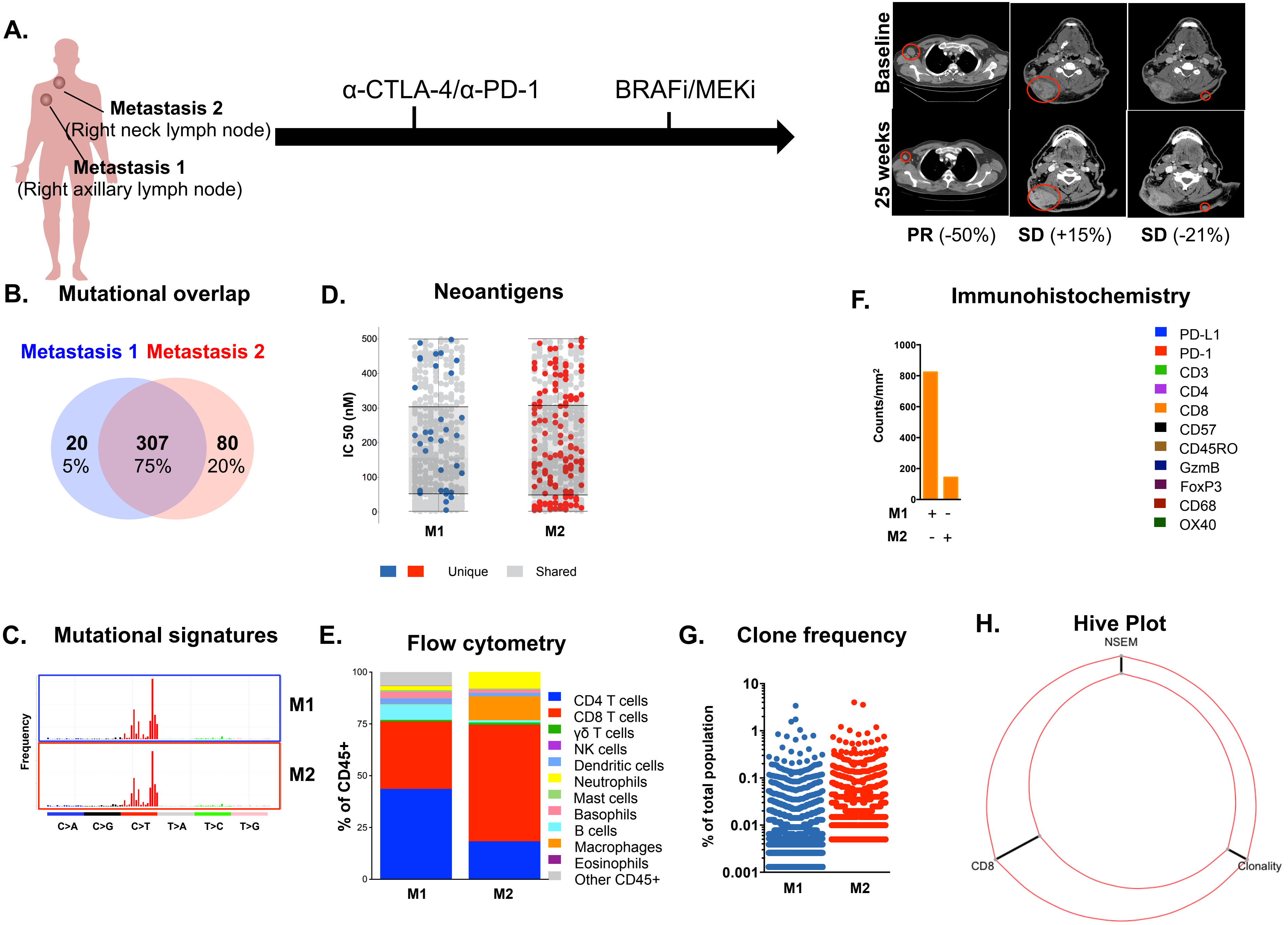
A.
Metastasis 2
(Right neck lymph node)
Metastasis 1
(Right axillary lymph node)
α-CTLA-4/α-PD-1
BRAFi/MEKi
Metastasis 1
Metastasis 2
Metastasis 3
Baseline
25 weeks
PR (-50%)
SD (+15%)
SD (-21%)
B.
Mutational overlap
Metastasis 1
Metastasis 2
20
5%
307
75%
80
20%
D.
Neoantigens
IC 50 (nM)
0
100
200
300
400
500
M1
M2
Unique
Shared
F.
Immunohistochemistry
Counts/mm²
0
200
400
600
800
1000
M1 + -
M2 - +
PD-L1
PD-1
CD3
CD4
CD8
CD57
CD45RO
GzmB
FoxP3
CD68
OX40
C.
Mutational signatures
Frequency
M1
M2
C>A
C>G
C>T
T>A
T>C
T>G
E.
Flow cytometry
% of CD45+
0
25
50
75
100
M1
M2
CD4 T cells
CD8 T cells
γδ T cells
NK cells
Dendritic cells
Neutrophils
Mast cells
Basophils
B cells
Macrophages
Eosinophils
Other CD45+
G.
Clone frequency
% of total population
0.001
0.01
0.1
1
10
M1
M2
H.
Hive Plot
NSEM
CD8
Clonality

Figure S5.

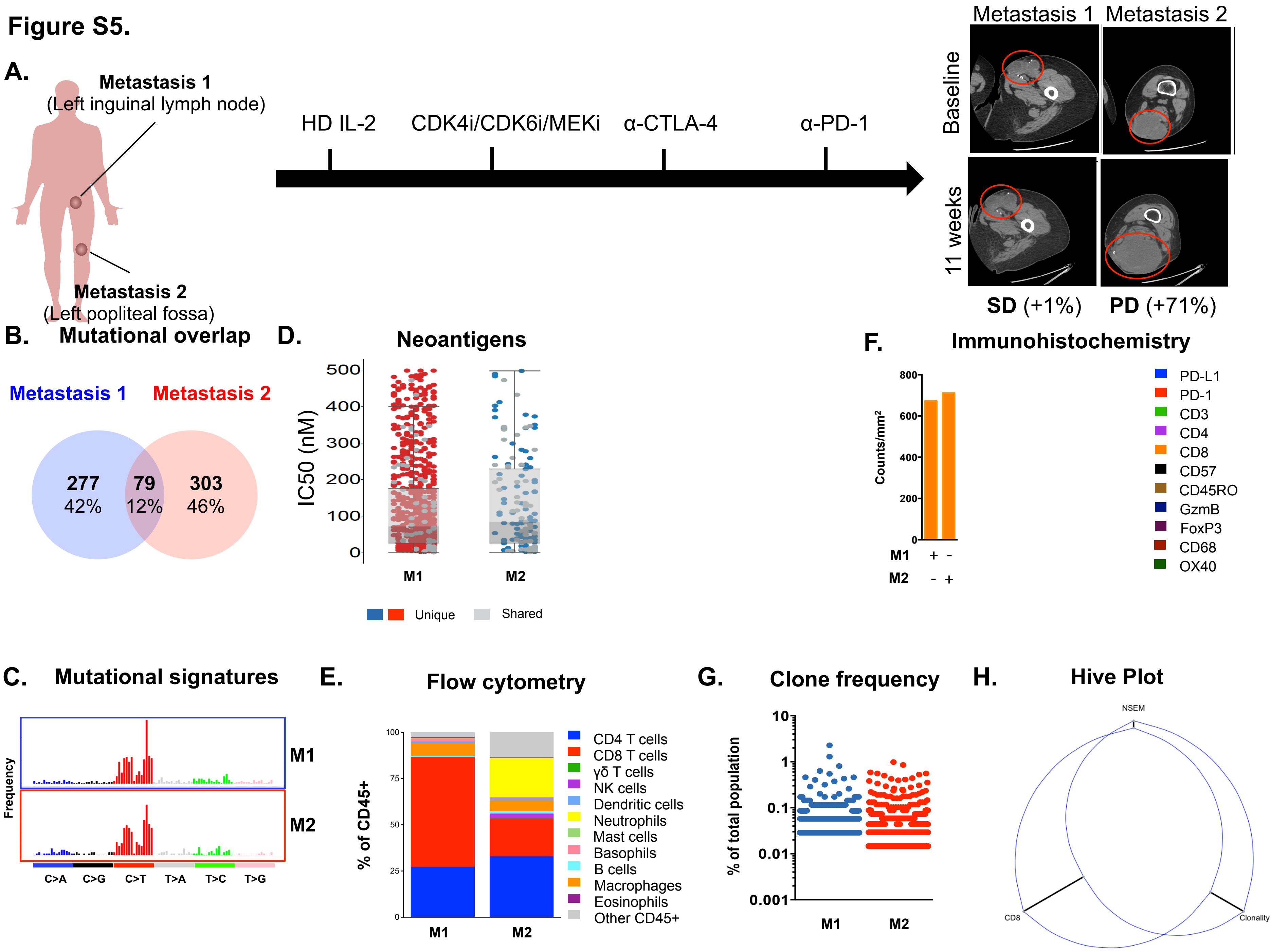
A.
Metastasis 1
(Left inguinal lymph node)
Metastasis 2
(Left popliteal fossa)
HD IL-2
CDK4i/CDK6i/MEKi
α-CTLA-4
α-PD-1
Metastasis 1
Metastasis 2
Baseline
11 weeks
SD (+1%)
PD (+71%)
B.
Mutational overlap
Metastasis 1
Metastasis 2
277
42%
79
12%
303
46%
C.
Mutational signatures
Frequency
M1
M2
C>A
C>G
C>T
T>A
T>C
T>G
D.
Neoantigens
IC50 (nM)
500
400
300
200
100
0
M1
M2
Unique
Shared
E.
Flow cytometry
% of CD45+
100
75
50
25
0
M1
M2
CD4 T cells
CD8 T cells
γδ T cells
NK cells
Dendritic cells
Neutrophils
Mast cells
Basophils
B cells
Macrophages
Eosinophils
Other CD45+
F.
Immunohistochemistry
Counts/mm²
800
600
400
200
0
M1 + -
M2 - +
PD-L1
PD-1
CD3
CD4
CD8
CD57
CD45RO
GzmB
FoxP3
CD68
OX40
G.
Clone frequency
% of total population
10
1
0.1
0.01
0.001
M1
M2
H.
Hive Plot
NSEM
CD8
Clonality

Figure S6.

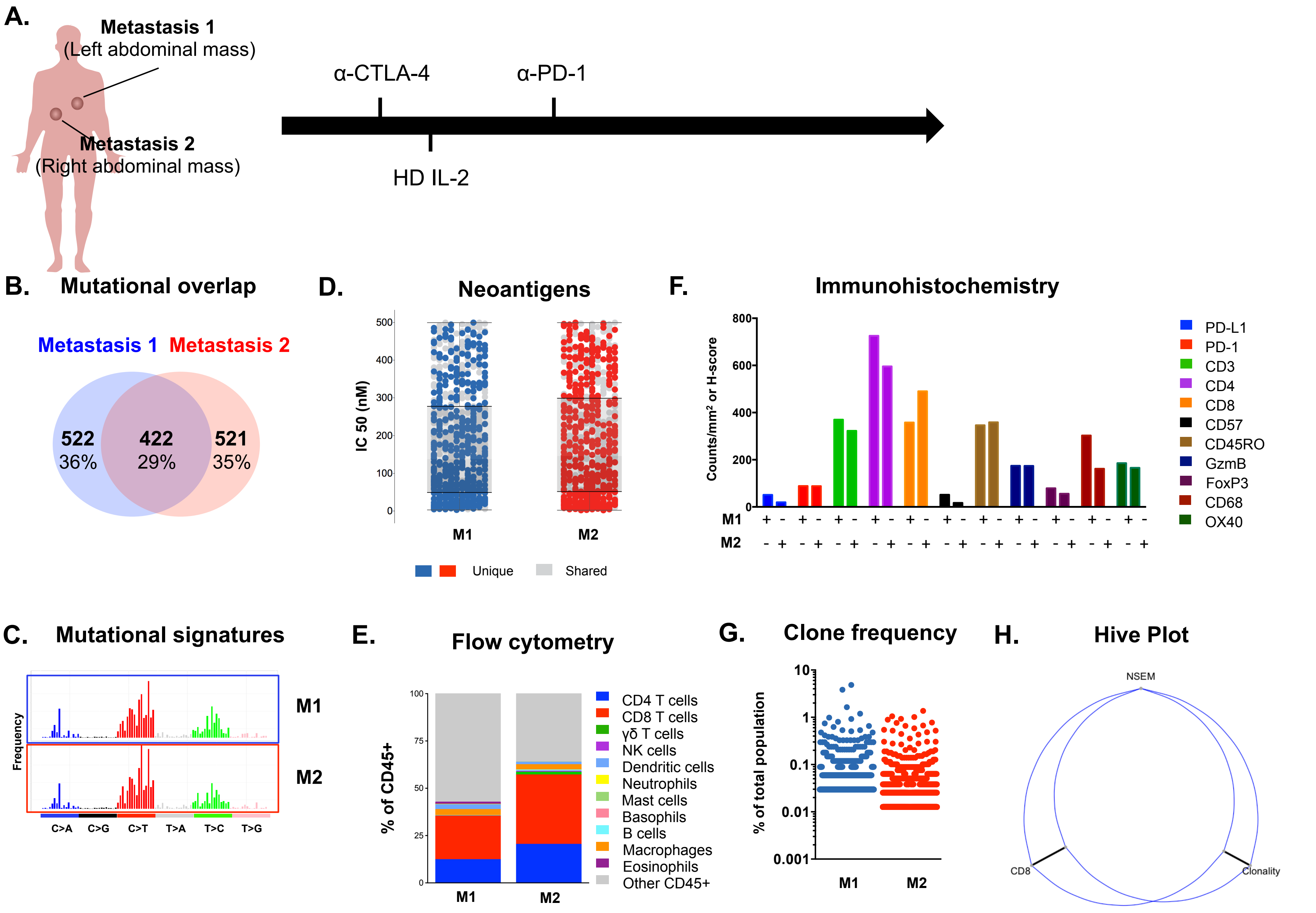

A.
Metastasis 1
(Left abdominal mass)
Metastasis 2
(Right abdominal mass)
α-CTLA-4
α-PD-1
HD IL-2
B. Mutational overlap
Metastasis 1
Metastasis 2
522
36%
422
29%
521
35%
D. Neoantigens
IC 50 (nM)
M1
M2
Unique
Shared
F. Immunohistochemistry
Counts/mm² or H-score
PD-L1
PD-1
CD3
CD4
CD8
CD57
CD45RO
GzmB
FoxP3
CD68
OX40
M1
M2
C. Mutational signatures
Frequency
M1
M2
C>A
C>G
C>T
T>A
T>C
T>G
E. Flow cytometry
% of CD45+
CD4 T cells
CD8 T cells
γδ T cells
NK cells
Dendritic cells
Neutrophils
Mast cells
Basophils
B cells
Macrophages
Eosinophils
Other CD45+
G. Clone frequency
% of total population
H. Hive Plot
NSEM
CD8
Clonality

Figure S7.

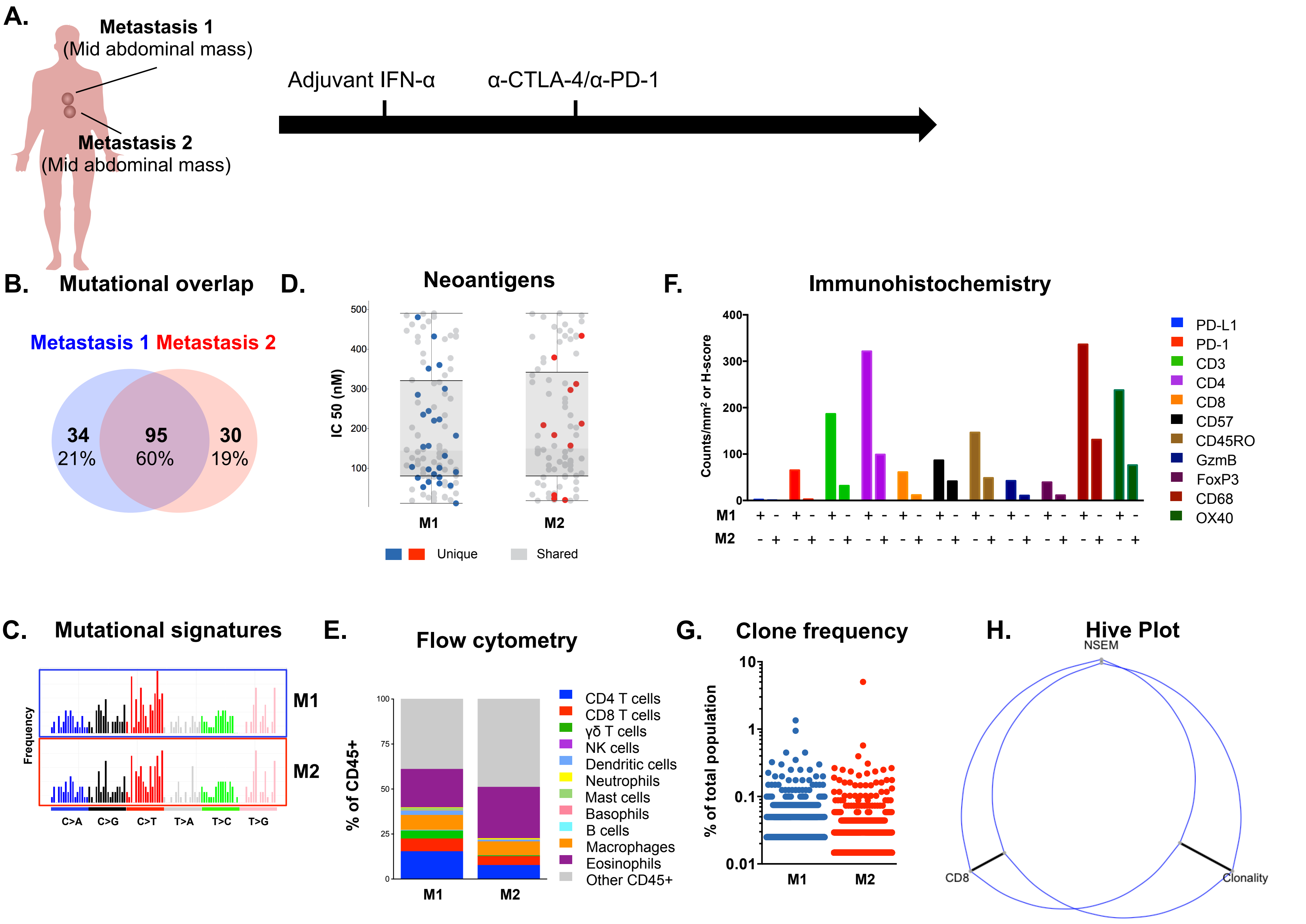
A.
Metastasis 1
(Mid abdominal mass)
Metastasis 2
(Mid abdominal mass)
Adjuvant IFN-α
α-CTLA-4/α-PD-1
B. Mutational overlap
Metastasis 1 Metastasis 2
34
21%
95
60%
30
19%
C. Mutational signatures
Frequency
M1
M2
C>A C>G C>T T>A T>C T>G
D. Neoantigens
IC 50 (nM)
M1 M2
Unique Shared
E. Flow cytometry
% of CD45+
M1 M2
CD4 T cells
CD8 T cells
γδ T cells
NK cells
Dendritic cells
Neutrophils
Mast cells
Basophils
B cells
Macrophages
Eosinophils
Other CD45+
F. Immunohistochemistry
Counts/mm² or H-score
M1
M2
PD-L1
PD-1
CD3
CD4
CD8
CD57
CD45RO
GzmB
FoxP3
CD68
OX40
G. Clone frequency
% of total population
M1 M2
H. Hive Plot
NSEM
CD8
Clonality

Figure S8.

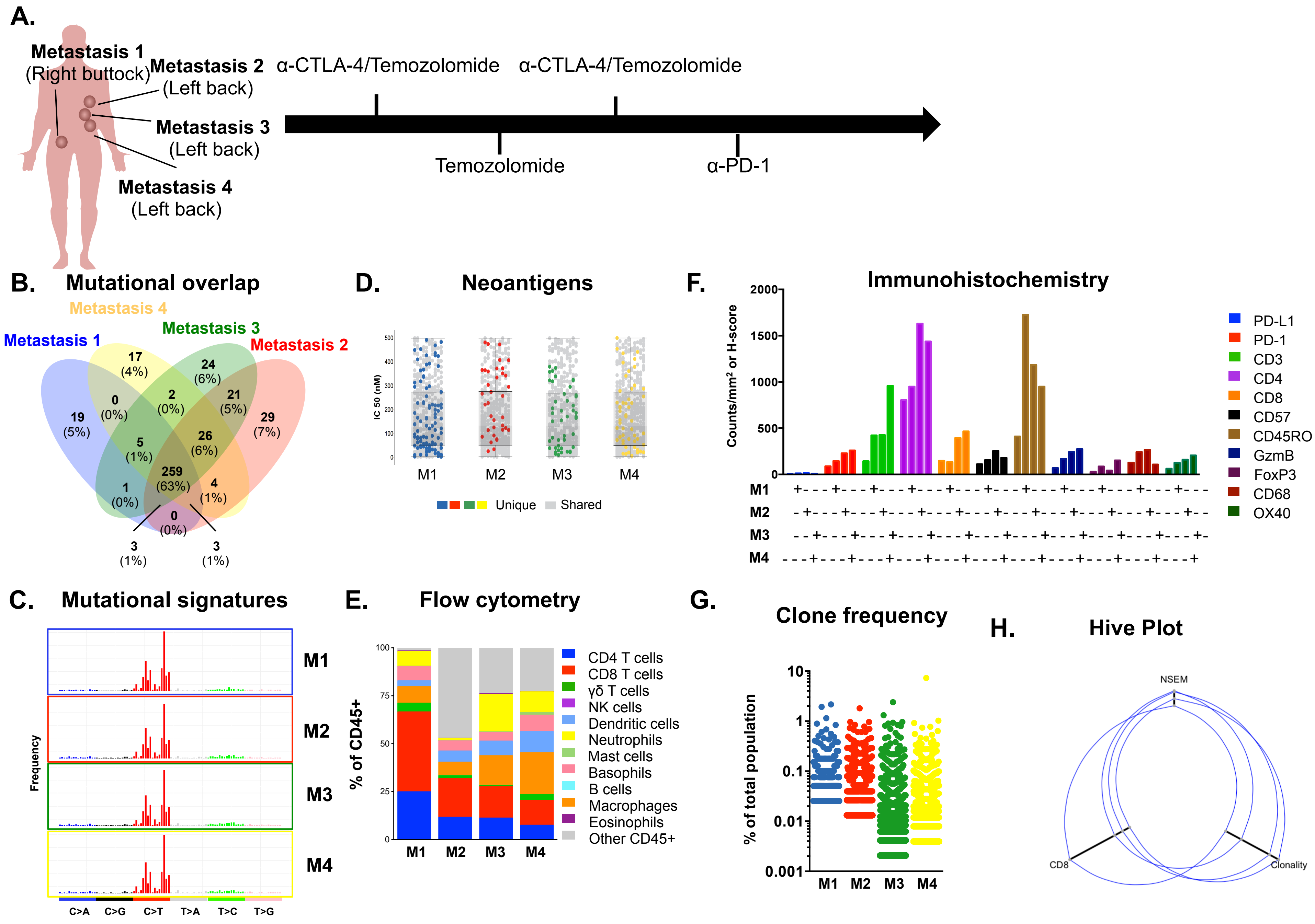
A.
Metastasis 1
(Right buttock)
Metastasis 2
(Left back)
Metastasis 3
(Left back)
Metastasis 4
(Left back)
α-CTLA-4/Temozolomide
α-CTLA-4/Temozolomide
Temozolomide
α-PD-1
B. Mutational overlap
Metastasis 4
Metastasis 3
Metastasis 1
Metastasis 2
17 (4%)
24 (6%)
0 (0%)
2 (0%)
21 (5%)
19 (5%)
29 (7%)
5 (1%)
26 (6%)
259 (63%)
1 (0%)
4 (1%)
0 (0%)
3 (1%)
3 (1%)
C. Mutational signatures
Frequency
M1
M2
M3
M4
C>A
C>G
C>T
T>A
T>C
T>G
D. Neoantigens
IC 50 (nM)
M1
M2
M3
M4
Unique
Shared
E. Flow cytometry
% of CD45+
M1
M2
M3
M4
CD4 T cells
CD8 T cells
γδ T cells
NK cells
Dendritic cells
Neutrophils
Mast cells
Basophils
B cells
Macrophages
Eosinophils
Other CD45+
F. Immunohistochemistry
Counts/mm² or H-score
M1
M2
M3
M4
PD-L1
PD-1
CD3
CD4
CD8
CD57
CD45RO
GzmB
FoxP3
CD68
OX40
G. Clone frequency
% of total population
M1
M2
M3
M4
H. Hive Plot
NSEM
CD8
Clonality

Figure S9.

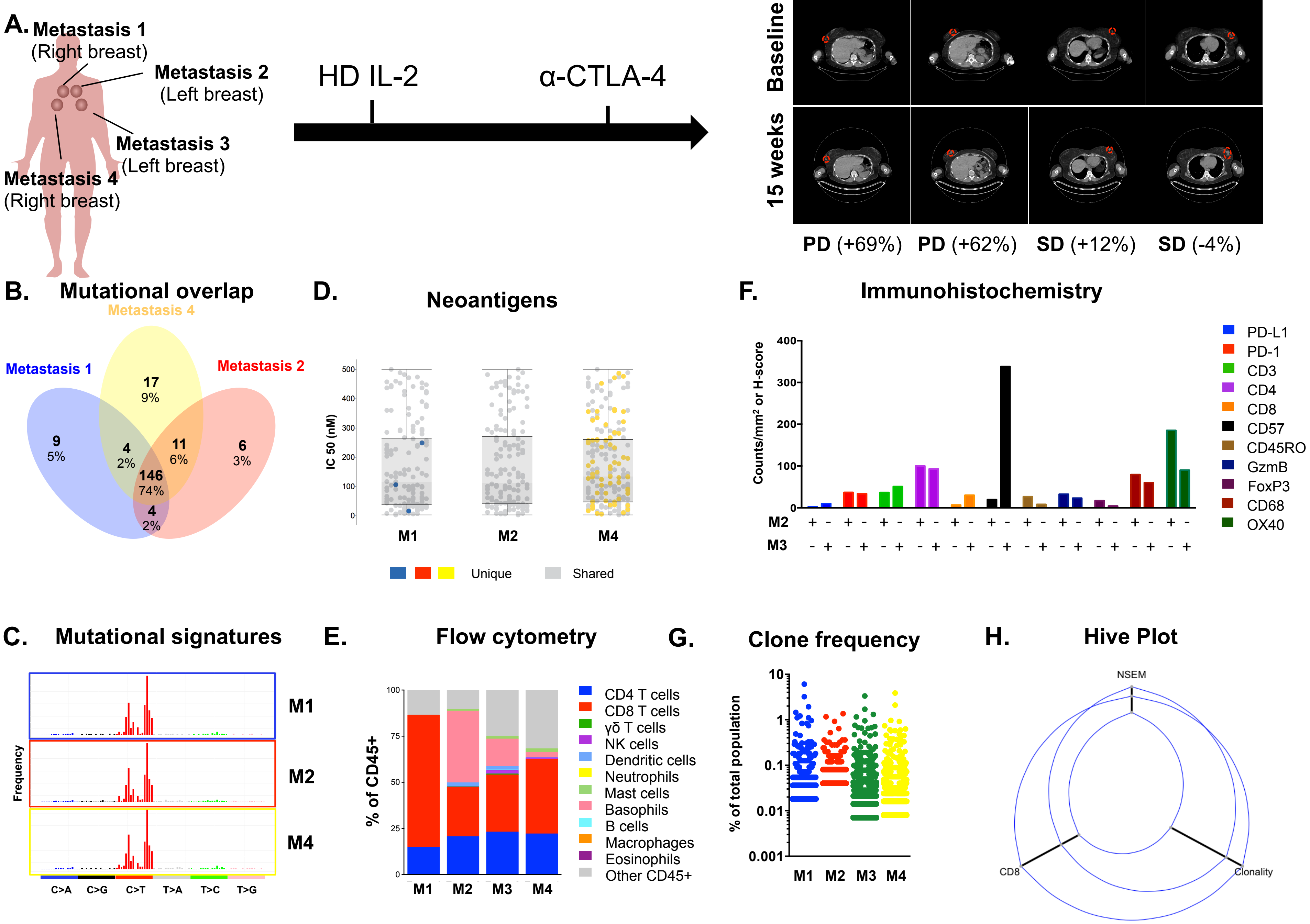
A.
Metastasis 1
(Right breast)
Metastasis 2
(Left breast)
Metastasis 3
(Left breast)
Metastasis 4
(Right breast)
HD IL-2
α-CTLA-4
Metastasis 1
Metastasis 2
Metastasis 3
Metastasis 4
Baseline
15 weeks
PD (+69%)
PD (+62%)
SD (+12%)
SD (-4%)
B.
Mutational overlap
Metastasis 4
Metastasis 1
Metastasis 2
17
9%
9
5%
4
2%
11
6%
6
3%
146
74%
4
2%
D.
Neoantigens
IC 50 (nM)
M1
M2
M4
Unique
Shared
F.
Immunohistochemistry
Counts/mm² or H-score
M2
M3
PD-L1
PD-1
CD3
CD4
CD8
CD57
CD45RO
GzmB
FoxP3
CD68
OX40
C.
Mutational signatures
Frequency
M1
M2
M4
C>A
C>G
C>T
T>A
T>C
T>G
E.
Flow cytometry
% of CD45+
CD4 T cells
CD8 T cells
γδ T cells
NK cells
Dendritic cells
Neutrophils
Mast cells
Basophils
B cells
Macrophages
Eosinophils
Other CD45+
G.
Clone frequency
% of total population
M1
M2
M3
M4
H.
Hive Plot
NSEM
CD8
Clonality

Figure S10.

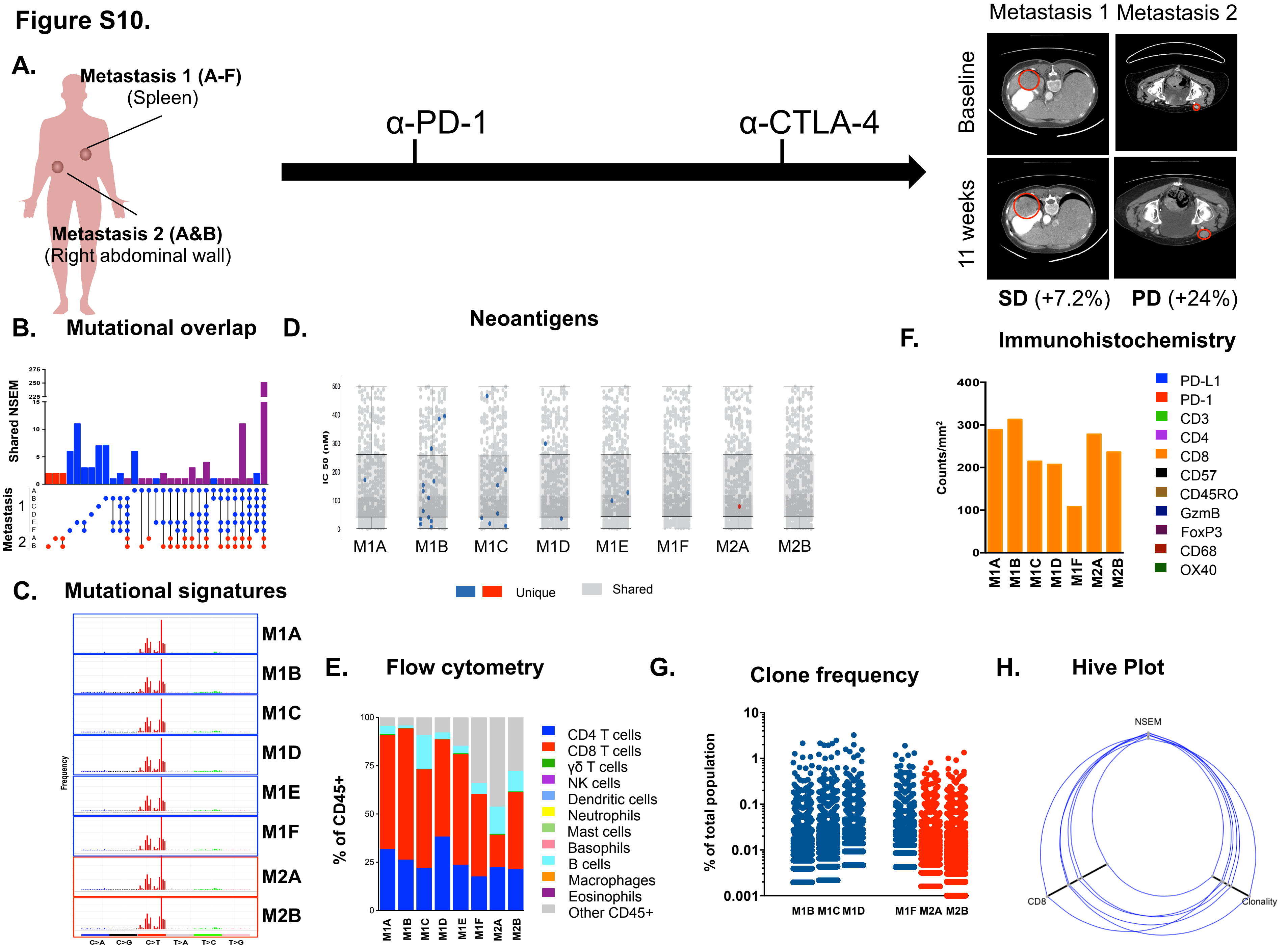
A.
Metastasis 1 (A-F)
(Spleen)
Metastasis 2 (A&B)
(Right abdominal wall)
α-PD-1
α-CTLA-4
Metastasis 1
Metastasis 2
Baseline
11 weeks
SD (+7.2%)
PD (+24%)
B.
Mutational overlap
Shared NSEM
Metastasis
D.
Neoantigens
IC 50 (nM)
M1A
M1B
M1C
M1D
M1E
M1F
M2A
M2B
Unique
Shared
F.
Immunohistochemistry
Counts/mm²
PD-L1
PD-1
CD3
CD4
CD8
CD57
CD45RO
GzmB
FoxP3
CD68
OX40
C.
Mutational signatures
Frequency
C>A
C>G
C>T
T>A
T>C
T>G
E.
Flow cytometry
% of CD45+
CD4 T cells
CD8 T cells
γδ T cells
NK cells
Dendritic cells
Neutrophils
Mast cells
Basophils
B cells
Macrophages
Eosinophils
Other CD45+
G.
Clone frequency
% of total population
M1B M1C M1D
M1F M2A M2B
H.
Hive Plot
NSEM
CD8
Clonality

Figure S11.

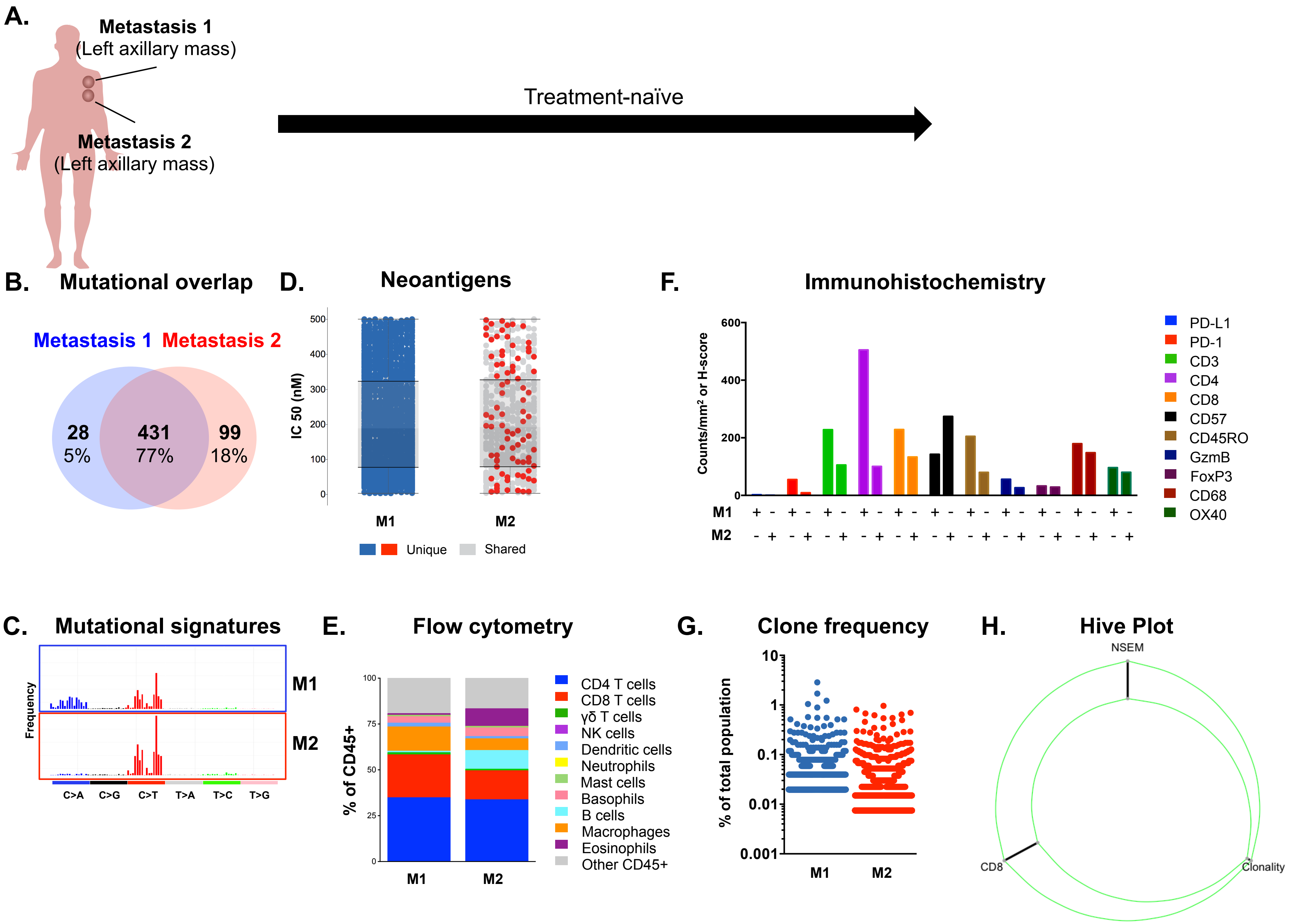
A.
Metastasis 1
(Left axillary mass)
Metastasis 2
(Left axillary mass)
Treatment-naïve
B. Mutational overlap
Metastasis 1 Metastasis 2
28
5%
431
77%
99
18%
C. Mutational signatures
Frequency
M1
M2
C>A C>G C>T T>A T>C T>G
D. Neoantigens
IC 50 (nM)
500
400
300
200
100
0
M1 M2
Unique Shared
E. Flow cytometry
% of CD45+
100
75
50
25
0
M1 M2
CD4 T cells
CD8 T cells
γδ T cells
NK cells
Dendritic cells
Neutrophils
Mast cells
Basophils
B cells
Macrophages
Eosinophils
Other CD45+
F. Immunohistochemistry
Counts/mm² or H-score
600
400
200
0
M1
M2
PD-L1
PD-1
CD3
CD4
CD8
CD57
CD45RO
GzmB
FoxP3
CD68
OX40
G. Clone frequency
% of total population
10
1
0.1
0.01
0.001
M1 M2
H. Hive Plot
NSEM
CD8
Clonality

Figure S12.

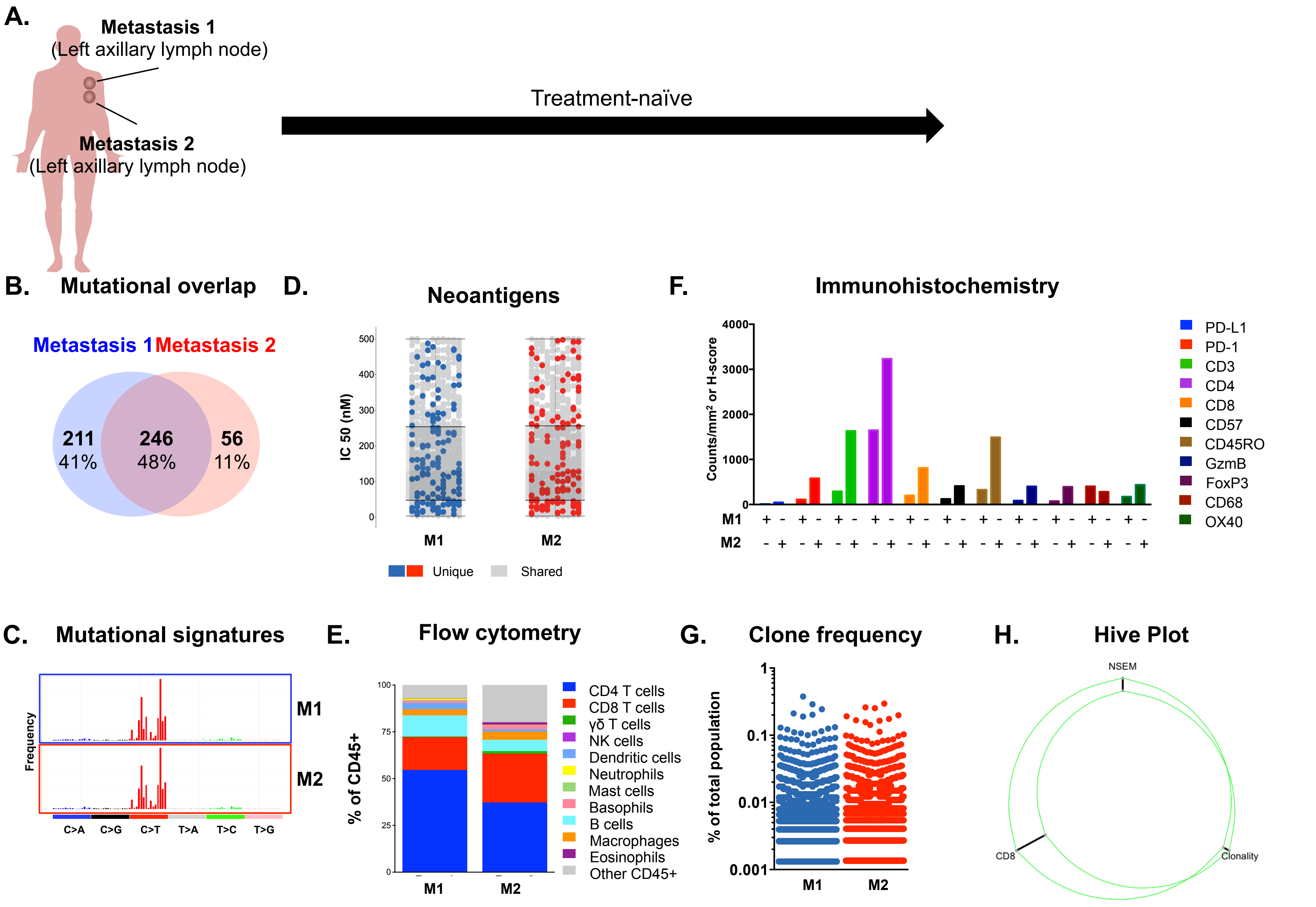
A.
Metastasis 1
(Left axillary lymph node)
Metastasis 2
(Left axillary lymph node)
Treatment-naïve
B.
Mutational overlap
Metastasis 1
Metastasis 2
211
41%
246
48%
56
11%
C.
Mutational signatures
Frequency
M1
M2
C>A
C>G
C>T
T>A
T>C
T>G
D.
Neoantigens
IC 50 (nM)
M1
M2
Unique
Shared
E.
Flow cytometry
% of CD45+
M1
M2
CD4 T cells
CD8 T cells
γδ T cells
NK cells
Dendritic cells
Neutrophils
Mast cells
Basophils
B cells
Macrophages
Eosinophils
Other CD45+
F.
Immunohistochemistry
Counts/mm² or H-score
M1
M2
PD-L1
PD-1
CD3
CD4
CD8
CD57
CD45RO
GzmB
FoxP3
CD68
OX40
G.
Clone frequency
% of total population
M1
M2
H.
Hive Plot
NSEM
CD8
Clonality

Figure S13.

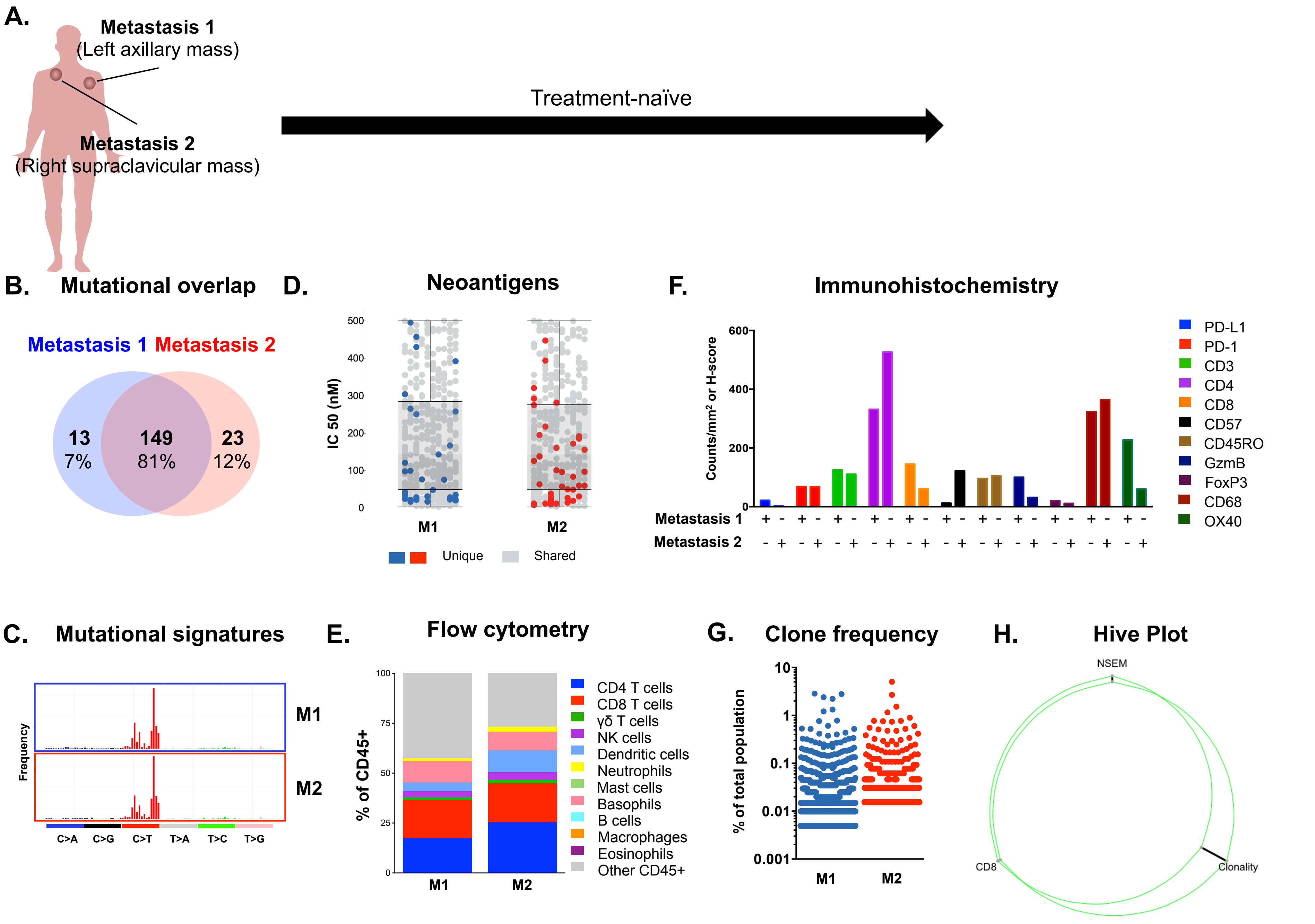

A.
Metastasis 1
(Left axillary mass)
Metastasis 2
(Right supraclavicular mass)
Treatment-naïve
B. Mutational overlap
Metastasis 1 Metastasis 2
13
7%
149
81%
23
12%
C. Mutational signatures
Frequency
M1
M2
C>A
C>G
C>T
T>A
T>C
T>G
D. Neoantigens
IC 50 (nM)
M1
M2
Unique
Shared
E. Flow cytometry
% of CD45+
M1
M2
CD4 T cells
CD8 T cells
γδ T cells
NK cells
Dendritic cells
Neutrophils
Mast cells
Basophils
B cells
Macrophages
Eosinophils
Other CD45+
F. Immunohistochemistry
Counts/mm² or H-score
Metastasis 1
Metastasis 2
PD-L1
PD-1
CD3
CD4
CD8
CD57
CD45RO
GzmB
FoxP3
CD68
OX40
G. Clone frequency
% of total population
M1
M2
H. Hive Plot
NSEM
CD8
Clonality

Figure S14.

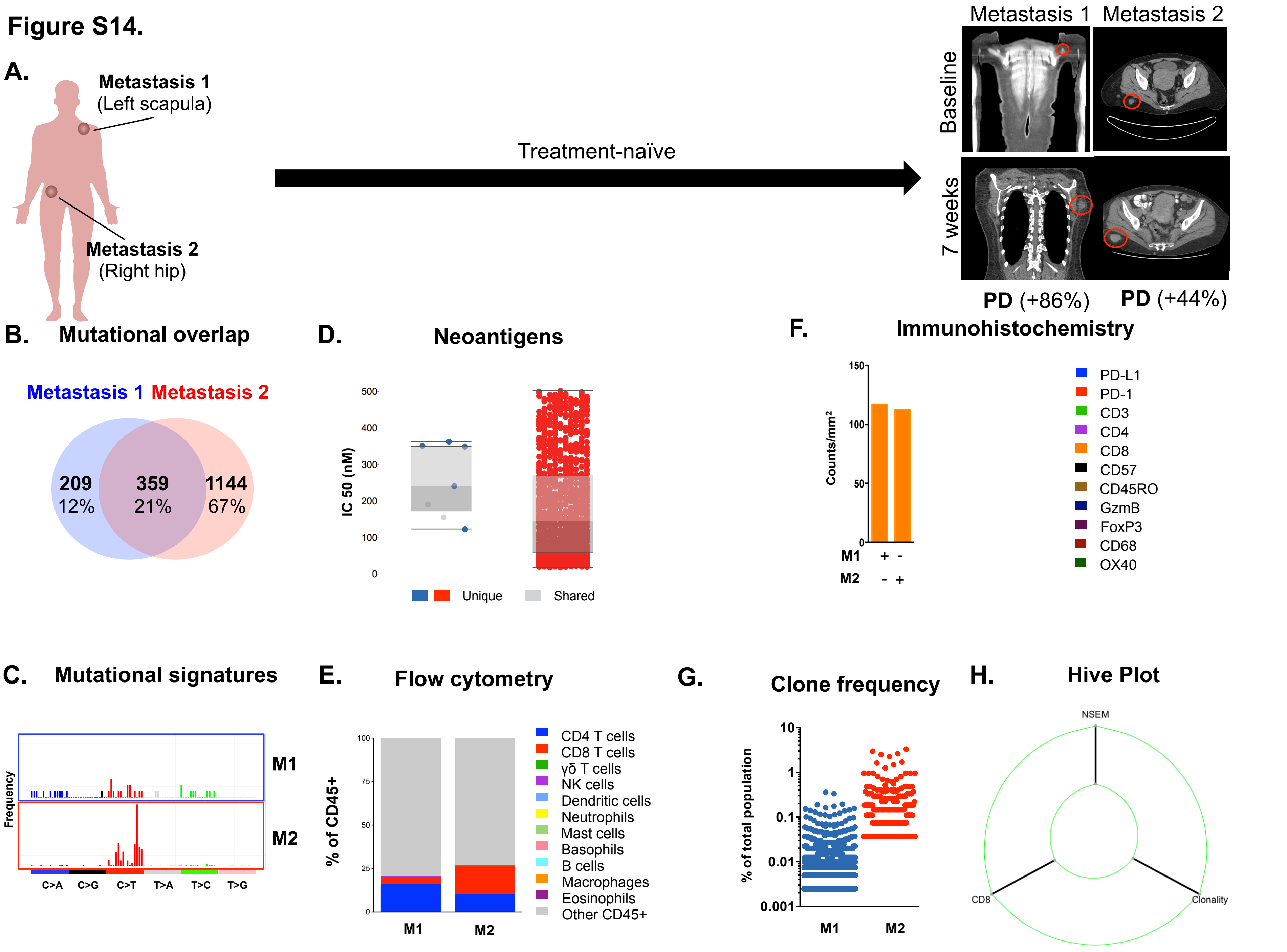
A.
Metastasis 1
(Left scapula)
Metastasis 2
(Right hip)
Treatment-naïve
Metastasis 1
Metastasis 2
Baseline
7 weeks
PD (+86%)
PD (+44%)
B.
Mutational overlap
Metastasis 1
Metastasis 2
209
12%
359
21%
1144
67%
C.
Mutational signatures
Frequency
M1
M2
C>A
C>G
C>T
T>A
T>C
T>G
D.
Neoantigens
IC 50 (nM)
0
100
200
300
400
500
Unique
Shared
E.
Flow cytometry
% of CD45+
0
25
50
75
100
M1
M2
CD4 T cells
CD8 T cells
γδ T cells
NK cells
Dendritic cells
Neutrophils
Mast cells
Basophils
B cells
Macrophages
Eosinophils
Other CD45+
F.
Immunohistochemistry
Counts/mm²
0
50
100
150
M1
M2
+
-
-
+
PD-L1
PD-1
CD3
CD4
CD8
CD57
CD45RO
GzmB
FoxP3
CD68
OX40
G.
Clone frequency
% of total population
0.001
0.01
0.1
1
10
M1
M2
H.
Hive Plot
NSEM
CD8
Clonality

Figure S15.

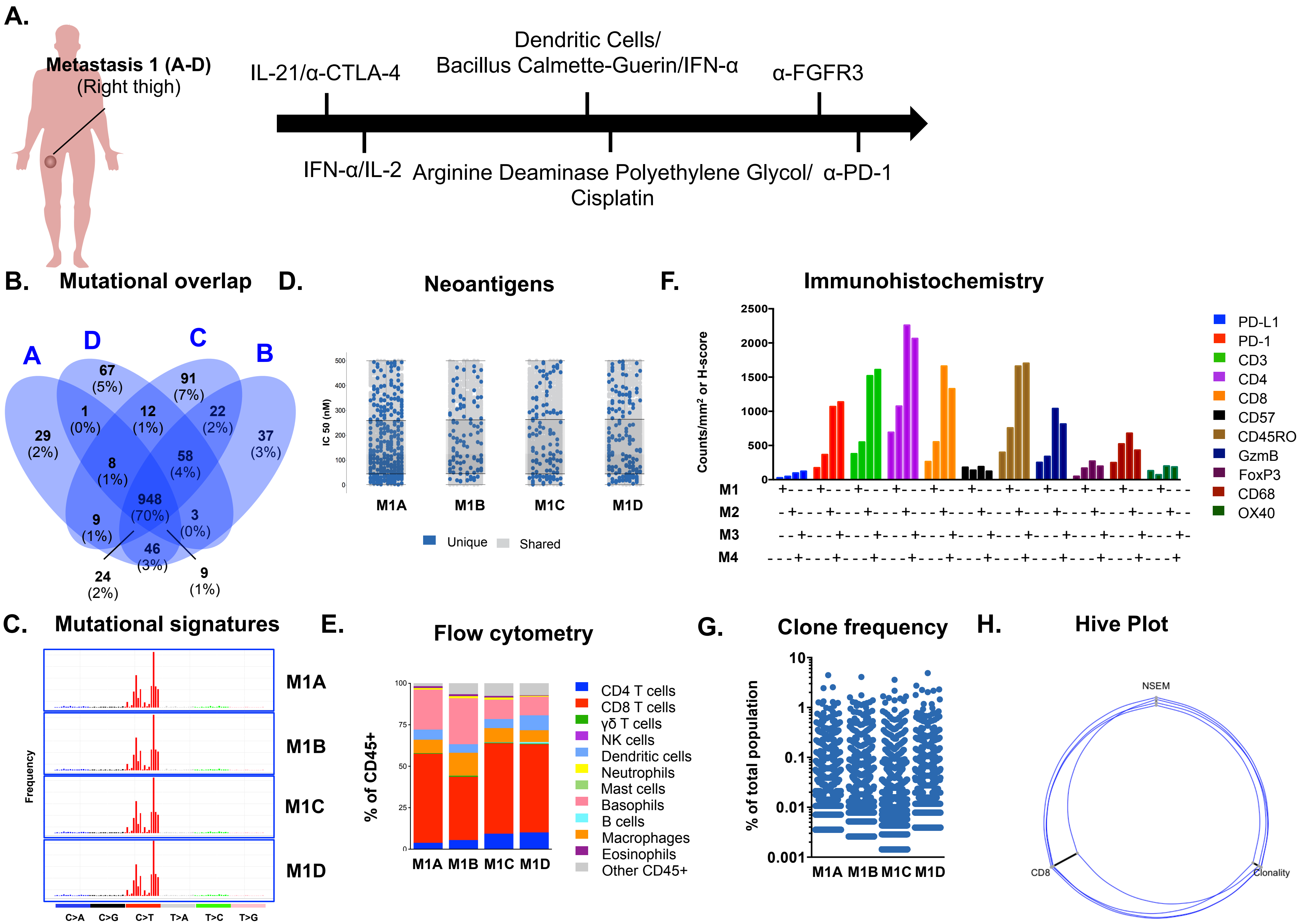
A.
Metastasis 1 (A-D)
(Right thigh)
IL-21/α-CTLA-4
Dendritic Cells/
Bacillus Calmette-Guerin/IFN-α
α-FGFR3
IFN-α/IL-2
Arginine Deaminase Polyethylene Glycol/
Cisplatin
α-PD-1
B. Mutational overlap
A
D
C
B
67
(5%)
91
(7%)
29
(2%)
1
(0%)
12
(1%)
22
(2%)
37
(3%)
8
(1%)
58
(4%)
948
(70%)
9
(1%)
3
(0%)
46
(3%)
24
(2%)
9
(1%)
D. Neoantigens
IC 50 (nM)
M1A
M1B
M1C
M1D
Unique
Shared
F. Immunohistochemistry
Counts/mm² or H-score
M1
M2
M3
M4
PD-L1
PD-1
CD3
CD4
CD8
CD57
CD45RO
GzmB
FoxP3
CD68
OX40
C. Mutational signatures
Frequency
M1A
M1B
M1C
M1D
C>A
C>G
C>T
T>A
T>C
T>G
E. Flow cytometry
% of CD45+
M1A
M1B
M1C
M1D
CD4 T cells
CD8 T cells
γδ T cells
NK cells
Dendritic cells
Neutrophils
Mast cells
Basophils
B cells
Macrophages
Eosinophils
Other CD45+
G. Clone frequency
% of total population
M1A
M1B
M1C
M1D
H. Hive Plot
NSEM
CD8
Clonality

Figure S16.

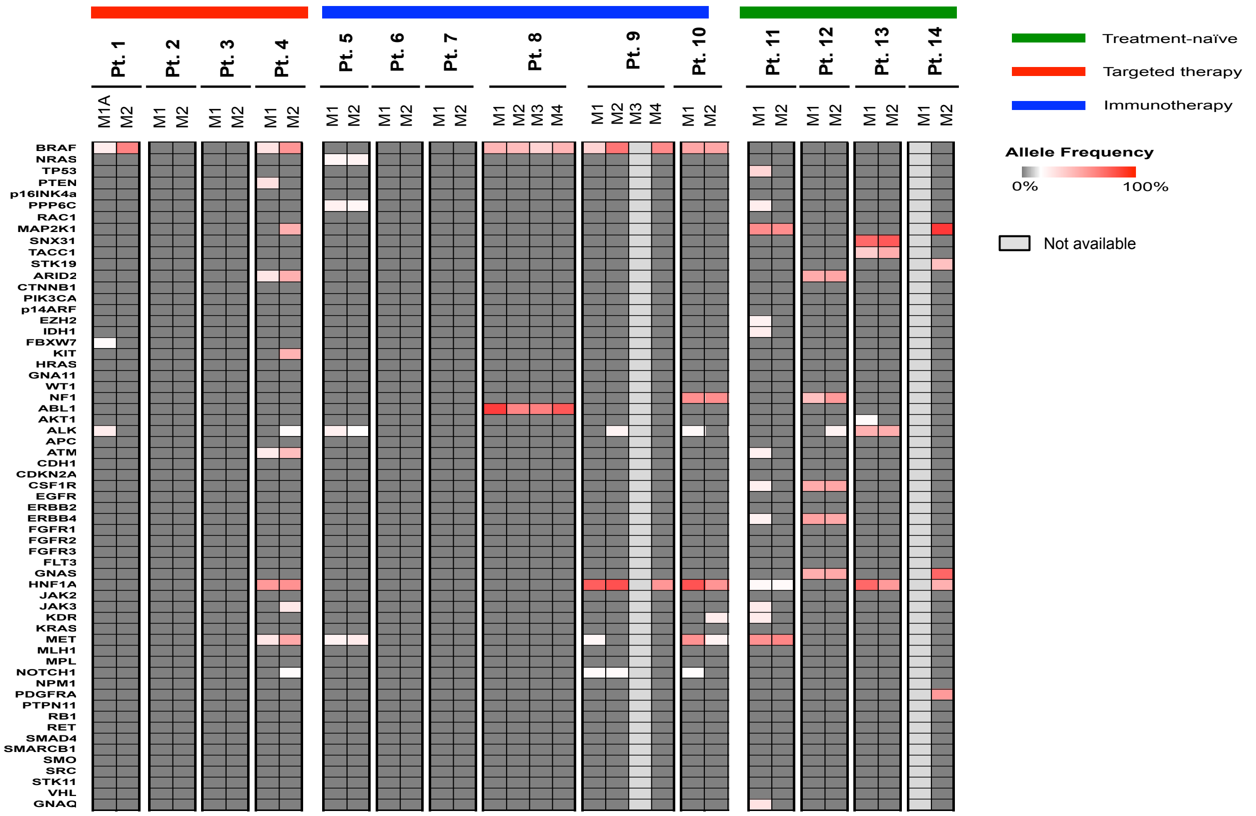
Pt. 1
Pt. 2
Pt. 3
Pt. 4
Pt. 5
Pt. 6
Pt. 7
Pt. 8
Pt. 9
Pt. 10
Pt. 11
Pt. 12
Pt. 13
Pt. 14
M1A
M1
M2
M3
M4
BRAF
NRAS
TP53
PTEN
p16INK4a
PPP6C
RAC1
MAP2K1
SNX31
TACC1
STK19
ARID2
CTNNB1
PIK3CA
p14ARF
EZH2
IDH1
FBXW7
KIT
HRAS
GNA11
WT1
NF1
ABL1
AKT1
ALK
APC
ATM
CDH1
CDKN2A
CSF1R
EGFR
ERBB2
ERBB4
FGFR1
FGFR2
FGFR3
FLT3
GNAS
HNF1A
JAK2
JAK3
KDR
KRAS
MET
MLH1
MPL
NOTCH1
NPM1
PDGFRA
PTPN11
RB1
RET
SMAD4
SMARCB1
SMO
SRC
STK11
VHL
GNAQ
Treatment-naïve
Targeted therapy
Immunotherapy
Allele Frequency
0%
100%
Not available

Figure S17.

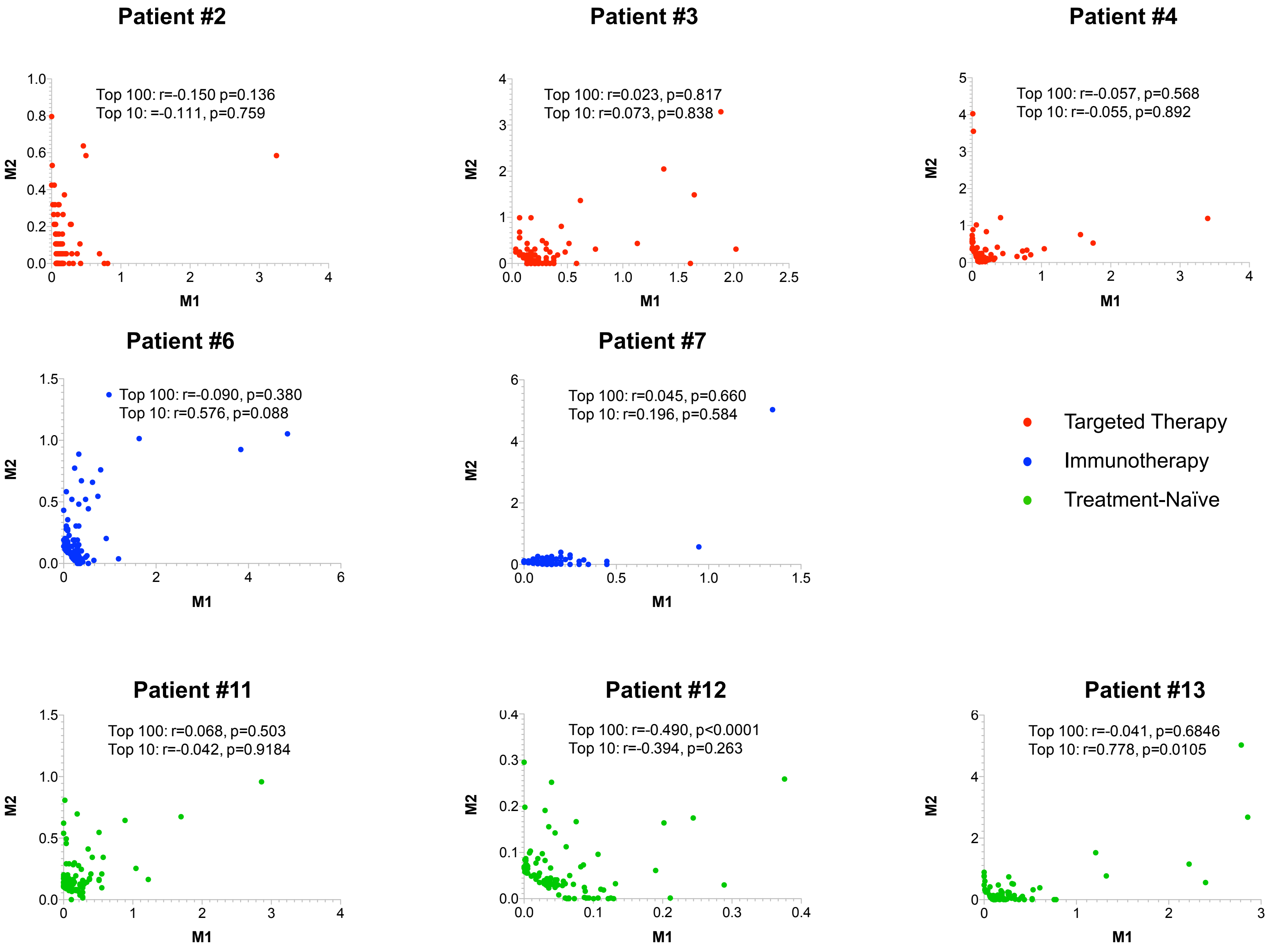
Patient #2
Top 100: r=-0.150 p=0.136
Top 10: =-0.111, p=0.759
Patient #3
Top 100: r=0.023, p=0.817
Top 10: r=0.073, p=0.838
Patient #4
Top 100: r=-0.057, p=0.568
Top 10: r=-0.055, p=0.892
Patient #6
Top 100: r=-0.090, p=0.380
Top 10: r=0.576, p=0.088
Patient #7
Top 100: r=0.045, p=0.660
Top 10: r=0.196, p=0.584
Targeted Therapy
Immunotherapy
Treatment-Naïve
Patient #11
Top 100: r=0.068, p=0.503
Top 10: r=-0.042, p=0.9184
Patient #12
Top 100: r=-0.490, p<0.0001
Top 10: r=-0.394, p=0.263
Patient #13
Top 100: r=-0.041, p=0.6846
Top 10: r=0.778, p=0.0105
M1
M2

Figure S18.

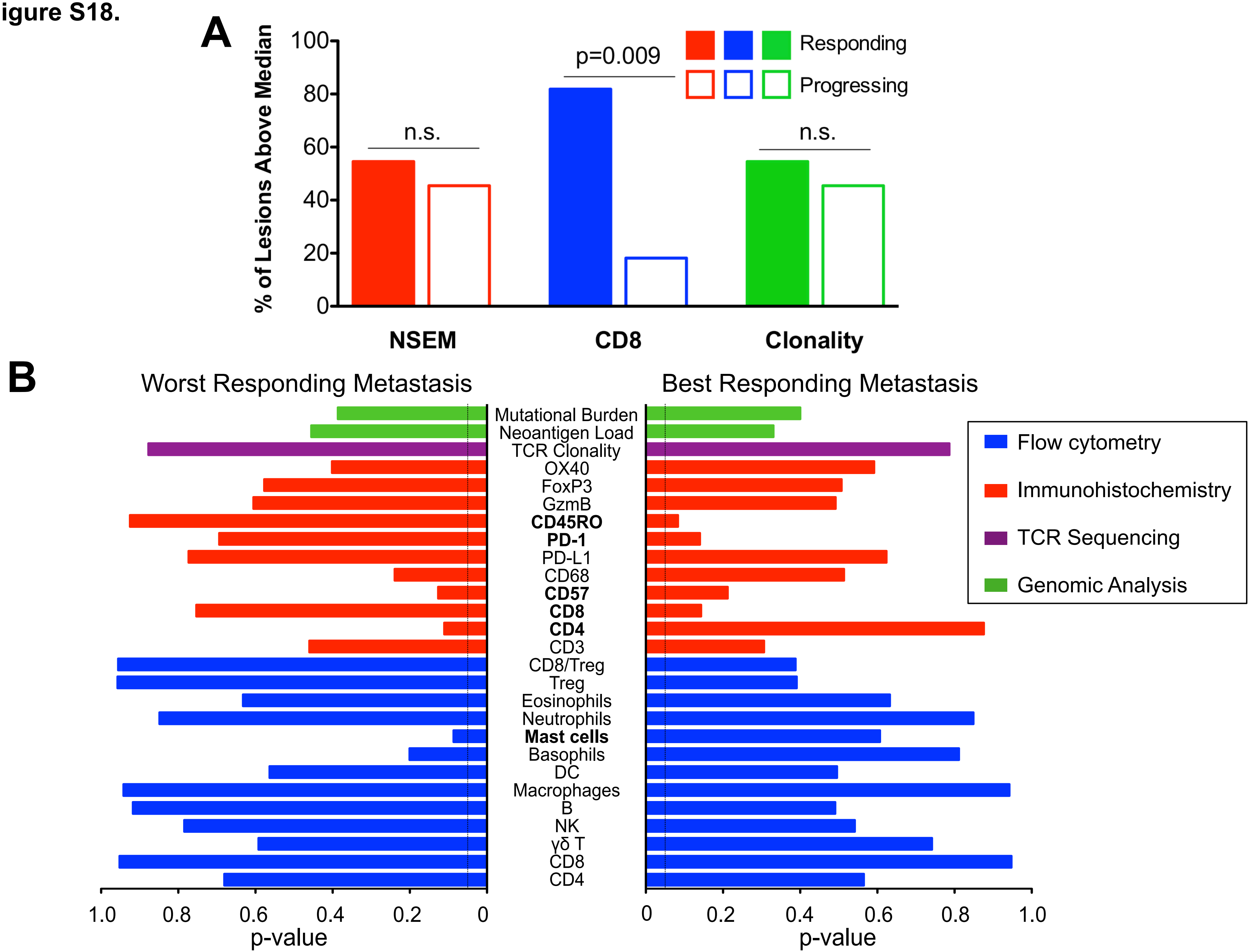
A
% of Lesions Above Median
100
80
60
40
20
0
n.s.
p=0.009
n.s.
Responding
Progressing
NSEM
CD8
Clonality
B
Worst Responding Metastasis
Best Responding Metastasis
Mutational Burden
Neoantigen Load
TCR Clonality
OX40
FoxP3
GzmB
CD45RO
PD-1
PD-L1
CD68
CD57
CD8
CD4
CD3
CD8/Treg
Treg
Eosinophils
Neutrophils
Mast cells
Basophils
DC
Macrophages
B
NK
γδ T
CD8
CD4
Flow cytometry
Immunohistochemistry
TCR Sequencing
Genomic Analysis
1.0
0.8
0.6
0.4
0.2
0
p-value
0
0.2
0.4
0.6
0.8
1.0
p-value

Figure S19.

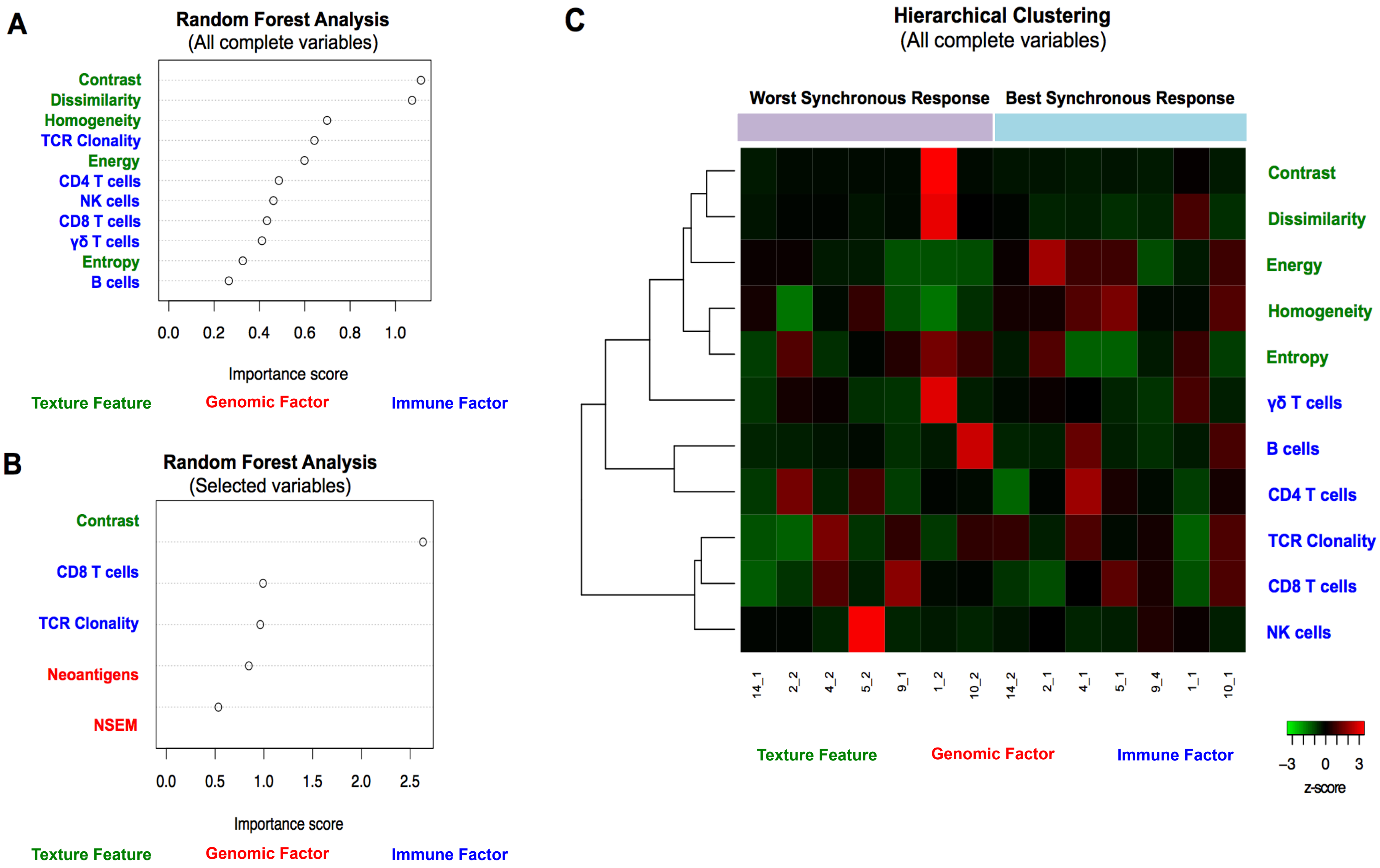
A
Random Forest Analysis
(All complete variables)
Contrast
Dissimilarity
Homogeneity
TCR Clonality
Energy
CD4 T cells
NK cells
CD8 T cells
γδ T cells
Entropy
B cells
0.0 0.2 0.4 0.6 0.8 1.0
Importance score
Texture Feature
Genomic Factor
Immune Factor
B
Random Forest Analysis
(Selected variables)
Contrast
CD8 T cells
TCR Clonality
Neoantigens
NSEM
0.0 0.5 1.0 1.5 2.0 2.5
Importance score
Texture Feature
Genomic Factor
Immune Factor
C
Hierarchical Clustering
(All complete variables)
Worst Synchronous Response
Best Synchronous Response
Contrast
Dissimilarity
Energy
Homogeneity
Entropy
γδ T cells
B cells
CD4 T cells
TCR Clonality
CD8 T cells
NK cells
14_1 2_2 4_2 5_2 9_1 1_2 10_2 14_2 2_1 4_1 5_1 9_4 1_1 10_1
Texture Feature
Genomic Factor
Immune Factor
−3 0 3
z-score

Figure S20.

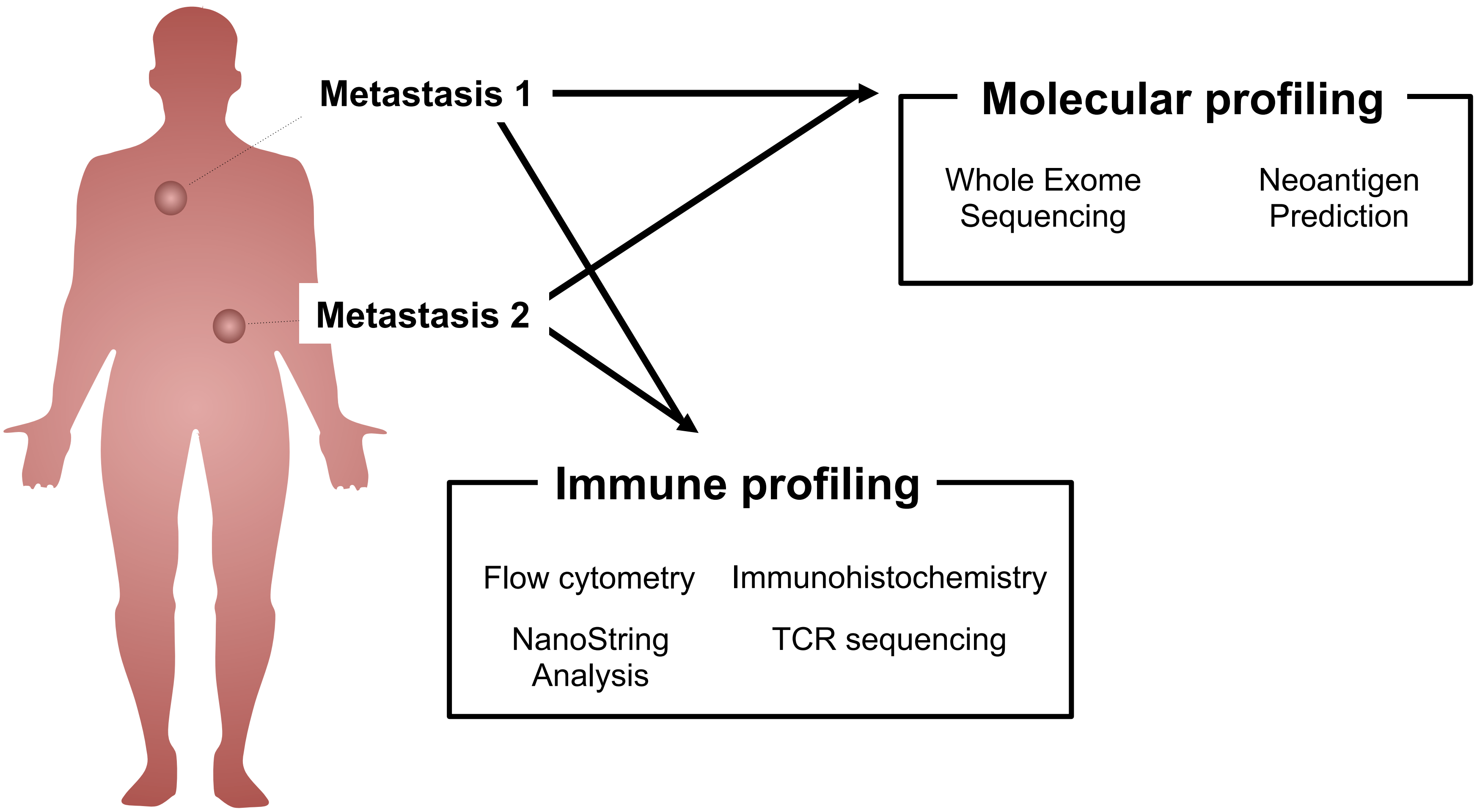

Metastasis 1
Metastasis 2
Molecular profiling
Whole Exome Sequencing
Neoantigen Prediction
Immune profiling
Flow cytometry
Immunohistochemistry
NanoString Analysis
TCR sequencing

Figure S21.

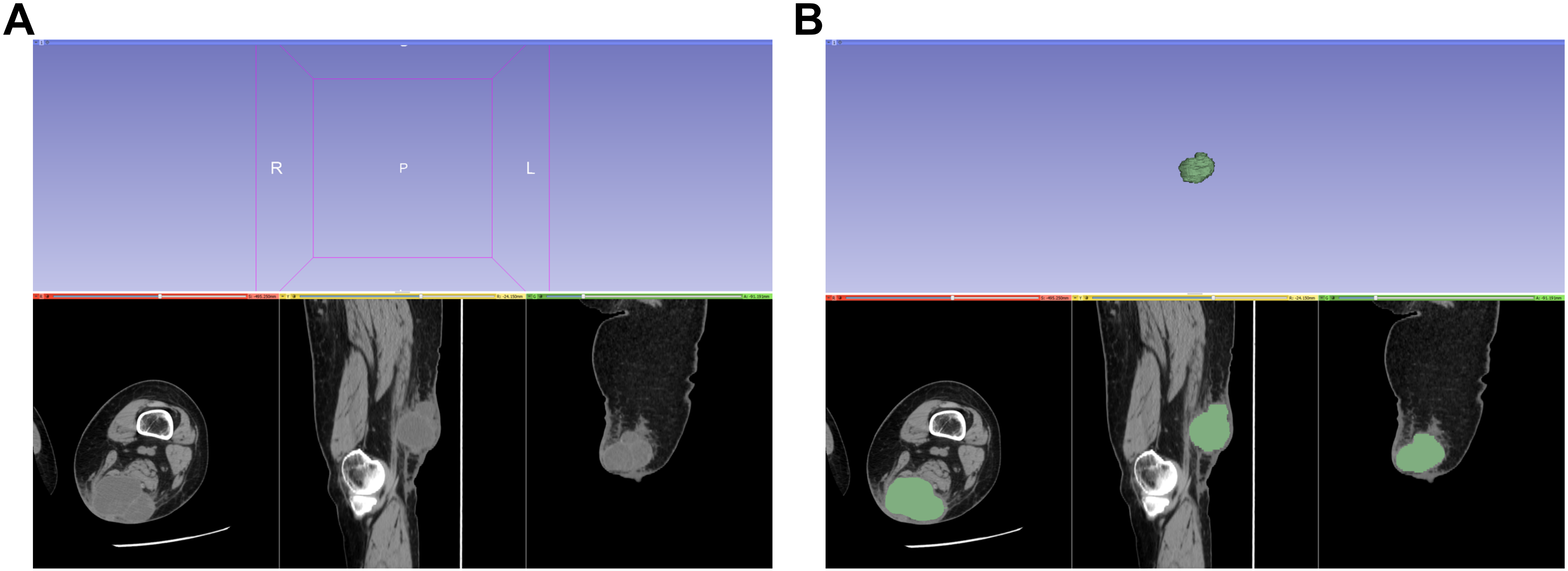

Supplement: Supplementary file 2 — Supplemental Figures S1-S21 [file 41525_2017_13_MOESM2_ESM.pdf]
