# Supplementary material for: Genomic and immune heterogeneity are associated with differential responses to therapy in melanoma
Source: NPJ Genom Med. 2017 Apr 7;2:10. doi: 10.1038/s41525-017-0013-8 (PMC5557036; doi:10.1038/s41525-017-0013-8)
Supplement: Supplementary file 3 — Supplemental Table Legends [file 41525_2017_13_MOESM3_ESM.docx]

**Supplementary Table Legends**

**Table S1. Radiographic responses in patients receiving first line targeted therapy or immune checkpoint blockade. A)** Description of patient cohort, age and gender, anatomical sites, % change in tumor size from baseline and RECIST class in 30 patients receiving first line BRAF/MEK-inhibitors. **B)** Description of patient cohort, age and gender, anatomical sites, % change in tumor size from baseline and RECIST class in 30 patients receiving first line PD-1 checkpoint blockade.

**Table S2. Proportion of patients experiencing heterogeneous responses to therapy.** Number and proportion of patients experiencing heterogeneous responses to therapy based on a threshold of 10%, 20%, and 50% in rate of tumor growth, as well as based on response classification and RECIST 1.1 class.

**Table S3. Patient demographics and characteristics.** Description of patient cohort, previous therapy, age and gender. Also included is the number of metastases evaluated, their anatomical sites, % change in tumor size from baseline and RECIST class, as well as HLA-A alleles for targeted therapy (n=4), immune checkpoint blockade (n=7), and treatment-naïve patients (n=4).

**Table S4. Molecular heterogeneity in synchronous melanoma metastases.** Aggregate genomic data showing number of somatic NSEM, percent unique and shared, relative contribution of mutational signatures to total mutational burden and neoantigen number and overlap in each sample. Grey, Low coverage sample. Green bar = treatment-naïve patients, red bar = targeted therapy patients, blue bar = immunotherapy patients.

**Table S5.** **Studies describing the genomic landscape in melanoma and genomic heterogeneity in synchronous tumors in melanoma, lung cancer, kidney cancer, and colorectal cancer.** Shown are a list of studies describing the mutational landscape in melanoma, as well as evaluating genomic comparisons between synchronous tumors in other tumor types, as well as their key findings relevant to our study.

**Table S6. Immune heterogeneity in synchronous melanoma metastases.** Aggregate flow cytometry data for all patients showing relative contribution of immune subsets as a percentage of total CD45+ cells. Green = treatment-naïve patient, red = targeted therapy patient, blue = immunotherapy patient. TCR clonality and number of unique T cell clones between samples within the same patient in the top 5%, 2.5%, 1%, and 0.5% most frequent TCRs detected. Grey, unavailable.

**Table S7. Gene expression profiling of synchronous melanoma metastases.** Gene expression profiling of synchronous by NanoString analysis of 795 cancer pathway- and immune-related genes within synchronous metastases. Values were normalized for each gene. Red, high expression; Blue, low expression; Black, undetectable.
